# Volume-outcome relationship on survival and cost benefits in severe burn injury: a retrospective analysis of a Japanese nationwide administrative database

**DOI:** 10.1186/s40560-019-0363-7

**Published:** 2019-01-30

**Authors:** Akira Endo, Atsushi Shiraishi, Yasuhiro Otomo, Kiyohide Fushimi, Kiyoshi Murata

**Affiliations:** 1 Trauma and Acute Critical Care Medical Center, Hospital of Medicine, Tokyo Medical and Dental University, 1-5-45 Yushima, Bunkyo-ku, Tokyo, 113-8510 Japan; 2 Emergency and Trauma Center, Kameda Medical Center, 929 Higashicho, Kamogawa, Chiba Japan; 3 Department of Health Policy and Informatics, Graduate School of Medicine, Tokyo Medical and Dental University, 1-5-45 Yushima, Bunkyo-ku, Tokyo, Japan; 4 The Shock Trauma and Emergency Medical Center, Matsudo City General Hospital, 933-1 Sendabori,, Matsudo, Chiba Japan

**Keywords:** Hospital volume, Costs and cost analysis, Mortality, Wounds and injuries

## Abstract

**Background:**

Although it has been reported that high hospital patient volume results in survival and cost benefits for several diseases, it is uncertain whether this association is applicable in burn care.

**Methods:**

We conducted a retrospective observational study on severe burn patients, defined by a burn index ≥ 10, using 2010–2015 data from a Japanese national administrative claim database. A generalized additive mixed-effect model (GAMM) was used to evaluate the nonlinear associations between patient volume and the outcomes (in-hospital mortality, healthcare costs per admission, and hospital-free days at 90 days). Generalized linear mixed-effect regression models (GLMMs) in which patient volume was incorporated as a continuous or categorical variable (≤ 5 or > 5) were also performed. Patient severity was adjusted using the prognostic burn index (PBI) or the risk adjustment model developed in this study, simultaneously controlling for hospital-level clustering. Sensitivity analyses evaluating patients who were directly transported, those with PBI ≤ 120 and those excluding patients who died within 2 days of admission, were also performed.

**Results:**

We analyzed 5250 eligible severe burn patients from 737 hospitals. The PBI and the developed risk adjustment model had good discriminative ability with areas under the receiver operating characteristic curves of 0.86 and 0.89, respectively. The GAMM plots showed that in-hospital mortality and healthcare costs increased according to the increase in patient volumes; then, they reached a plateau. Fewer hospital-free days were observed in the higher volume hospitals. The GLMM model showed that patient volume (incorporated as a continuous variable) was significantly associated with increased in-hospital mortality (adjusted odds ratio [95% confidence interval (CI)] = 1.14 [1.09–1.19]), high healthcare costs (adjusted difference [95% CI] = $4876 [4436–5316]), and few hospital-free days (adjusted difference [95% CI] = − 3.1 days [− 3.4 to − 2.8]). Similar trends were observed in the analyses in which patient volume was incorporated as a categorical variable. The results of sensitivity analyses showed comparable results.

**Conclusions:**

Analysis of Japanese nationwide administrative database demonstrated that high burn patient volume was significantly associated with increased in-hospital mortality, high healthcare costs, and few hospital-free days. Further studies are needed to validate our results.

**Electronic supplementary material:**

The online version of this article (10.1186/s40560-019-0363-7) contains supplementary material, which is available to authorized users.

## Introduction

Although the incidence, severity, and mortality rates of burns were reported to be decreasing in highly developed countries, burns still generate a significant socioeconomic and healthcare burden globally [1, 2]. A potential volume-outcome relationship has been reported in several diseases or procedures requiring highly specialized care or techniques [3–5]. Treatment for severe burn injury generally requires enormous amounts of human and material resources relating to specialized intensive care, multistage surgery, and prolonged rehabilitation. Therefore, it was recommended that severe burn injury should be treated by expert multidisciplinary teams that are skilled in specialized strategy [6]. Hence, we hypothesized that a volume-outcome relationship can be anticipated in severe burn care, and thus, superiority regarding patient outcomes and resource utilization is expected in high-volume facilities. However, the results from existing literatures that investigated this relationship in burn patients were inconsistent, with some studies reporting associations between a high patient volume and reductions in in-hospital mortality [7, 8], and other studies reporting no such association [9–11]. Furthermore, most of those studies included less severe cases not requiring specialized strategy. Studies regarding healthcare costs according to patient volume have been scarce.

In this study, we evaluated the associations of hospitals’ severe burn patient volume with survival and cost benefits using a Japanese national administrative inpatient database.

## Methods

### Study design and settings

This was a retrospective study that evaluated the association between hospital patient volume and outcomes in severe burn patients. We analyzed the data of the Japanese Diagnosis Procedure Combination (DPC) database from the fiscal year of 2010 to 2015. The DPC database is a Japanese nationwide administrative database linked to the reimbursement system for inpatients in Japanese hospitals. It contains claims for every drug administered and every procedure and care provided for each patient during hospitalization. The payments can be estimated based on the reference prices in the Japanese fee schedule, which was recorded on a piecework payment basis rather than a bundled payment basis and independent from institutional functional evaluation coefficients. Furthermore, the DPC database also functions as a case-mix classification system. In addition to the patient demographic information and the treating hospital’s information, a maximum of four primary diagnoses, comorbidities at admission, and post-admission complications are independently recorded for each patient using the International Classification of Diseases, 10th Revision (ICD-10) code. Since more than 1500 hospitals including 271/279 (97.1%) of government-approved tertiary emergency hospitals had participated in the DPC database by the end of 2015, a substantial proportion of severe burn patients could be identified using the database. Further details regarding the DPC database were reviewed elsewhere [12].

This study was conducted according to the principles of the 1964 Declaration of Helsinki and its later amendments. The institutional review board of the Tokyo Medical and Dental University approved this study (#788). Informed consent from each patient was not required because of the retrospective study design and the use of anonymized patient and hospital data.

### Study population

Patients admitted because of burn injury (ICD-10 codes T20–T31) between April 1, 2010, and March 31, 2015, were identified in the DPC database. Of those, we included patients whose burn index [13] was more than or equal to 10. We excluded patients who had experienced cardiac arrest in prehospital settings, had the diagnosis of burn sequelae (T95X) at admission, and lacked the specific code for emergency admission. For patients who were admitted multiple times with the diagnostic code of burn, the second and subsequent admission(s) were excluded from the analysis. Patients who were transferred to another hospital within 3 days of admission were also excluded because most of them could be regarded as not having completed the initial management and were transferred for specialized care.

### Data collection

We collected the following information: age; sex; ICD-10 codes for primary diagnoses and concurrent diagnoses at admission; burn index; year of injury; unique hospital identifier; information regarding whether the patient was transferred from another hospital; information regarding whether patient had a disturbance of consciousness; information regarding presence of inhalation injury; presence or absence of specific reimbursement claims during the first 2 days of admission regarding escharotomy, mechanical ventilation, red blood cell (RBC) transfusion, haptoglobin, vasopressors (norepinephrine or dopamine), and intensive care provided by government-approved advanced intensive care units (ICUs); length of hospital stay; status at hospital discharge (survived or deceased); and total healthcare cost per admission. We further collected information on whether skin grafting was performed during hospitalization, in which the information on the use of artificial epidermis or autologous cultured epidermis were also collected to assess the treating hospital’s preference. Regarding hospital-related variables, information on the hospital status (whether it was a government-approved advanced hospital) and the number of ICU beds in the hospital were collected. Patient comorbidities were assessed using the Charlson Comorbidity Index [14] that was calculated by extracting the ICD-10 codes based on the method reported by Quan et al. [15]. The prognostic burn index (PBI) [16], the burn severity score widely used in Asian countries [16–19], was calculated for each patient using age and burn index.

### Definitions and outcomes

The primary outcome was defined as in-hospital mortality, and the secondary outcomes were defined as total healthcare costs and hospital-free days at 90 days. Patient volume was defined as the mean number of annual severe burn patients admitted in each hospital during the study period. Hospital-free days were defined as days of being alive and free from hospitalization, which were recommended as composite measures for the length of hospital stay and mortality [20]. Total healthcare costs were defined as all aggregated payments (except for boarding costs) during hospitalization, including costs for surgical, pharmacological, laboratory, and other inpatient services. As the costs were recorded in yen in the DPC database, in this analysis, we converted them into US dollars (100 yen = $1 USD).

### Statistical analysis

To appropriately estimate the patient volume per hospital, we used a multiple imputation method because the missing mechanism in the naïve data could be assumed as missing at random from clinical perspective. Missing data on the collected variables were complemented by multivariate imputation using chained equations with 10 iterations, and 15 datasets were produced. Descriptive statistics displayed categorical variables as counts and percentages, and numeric or ordered variables as medians and interquartile ranges (IQR) after pooling all the imputed datasets into one dataset. Predictive statistics displayed the estimators as point estimation and 95% confidence intervals (CI) integrated across the imputed datasets based on Rubin’s rule [21].

We evaluated the association between patient volume and the outcomes using a generalized additive mixed-effect model (GAMM) with the residual maximum likelihood method, considering possible nonlinear relationships between patient volume and outcomes [22]. The model was adjusted for PBI (a fixed effect variable) and the unique hospital identifier (a random effect variable), while patient volume was included in the model as a smoothing term. We further analyzed volume-outcome relationships using a generalized linear mixed model (GLMM), adjusted for PBI (a fixed effect variable) and the unique hospital identifier (a random effect variable). In this analysis, patient volume was included in the model as a continuous or dichotomized categorical variable (≤ 5 or > 5). The cutoff threshold was determined to maintain a sufficient number of patients and hospitals in each group. A mixed-effects logistic regression model was used for the outcome of in-hospital mortality, and a mixed-effect linear regression model was used for the outcomes of total healthcare costs and hospital-free days. GAMM and GLMM models could explain both patient-level confounding and hospital-level clustering simultaneously. Regarding the cost analyses, considering the effect of the length of the hospital stay, analyses adjusting for the length of hospital stay (as a fixed effect variable) were also performed.

We further performed sensitivity analyses by analyzing different sub-study populations. First, we analyzed the population who were directly transported to the hospital from the injured scene, considering the heterogeneity of patients transferred from another hospital. Second, we analyzed only the population with PBI ≤ 120, considering the possibility that the high-volume hospitals treated more number of fatal patients whom we could not rescue even if every high-quality treatment was provided. In these two analyses, the volume-outcome relationship was assessed using the aforementioned GAMM and GLMM models with the adjustment for PBI. Finally, considering the issue of residual confounding due to the nature of the retrospective study design, we developed a risk adjustment model for in-hospital mortality by including the following variables: age, sex, burn index, year of injury, Charlson Comorbidity Index, consciousness level at admission (alert or not), admitted ward, and presence or absence of inhalation injury, as well as the following interventions performed within 2 days of admission: mechanical ventilation, escharotomy, haptoglobin use, vasopressor use, and RBC transfusion. Hospital-related variables on the number of ICU beds, the hospital status, and the proportion of transferred patients from another hospital during the observation period were also included in the model. Those variables were selected based on previous studies [23, 24] and clinical perspective, and they were incorporated into a logistic regression model. Issues with variable multicollinearity were assessed by a variance inflation factor, and the tolerance value was set at lower than 2. In this model, we were forced to exclude patients who died within 2 days of admission considering the issue of immortal time bias, because variables for interventions performed within 2 days of admission were used in the developed risk adjustment model. The risk adjustment model was established using a random sample of 80% of the cohort, and its discriminative performance was validated in the remaining 20% of the cohort. Discriminative ability and calibration were assessed using the area under the receiver operating characteristic curve (AUROC) and a Hosmer-Lemeshow goodness-of-fit test, respectively. We then evaluated the association between patient volume and the outcomes using GAMM and GLMM models adjusted for the developed risk adjustment model instead of PBI.

As eliminating the issue of residual confounding was impossible because of the nature of the retrospective database analysis, we performed a quantitative bias analysis for in-hospital mortality in the primary analysis model. We evaluated an *E* value that can objectively estimate the minimum impact of a hypothetical unmeasured confounder, which can explain the observed risk away, without the assumption of prevalence of the unmeasured confounder. [25, 26]

All statistical analyses were performed using R software (version 3.5.1; R Foundation for Statistical Computing, Vienna, Austria) and a commander module that incorporates frequently used biostatistical functions. A two-sided *p* value < 0.05 was considered as statistically significant.

## Results

### Primary analysis

**Fig. 1:**
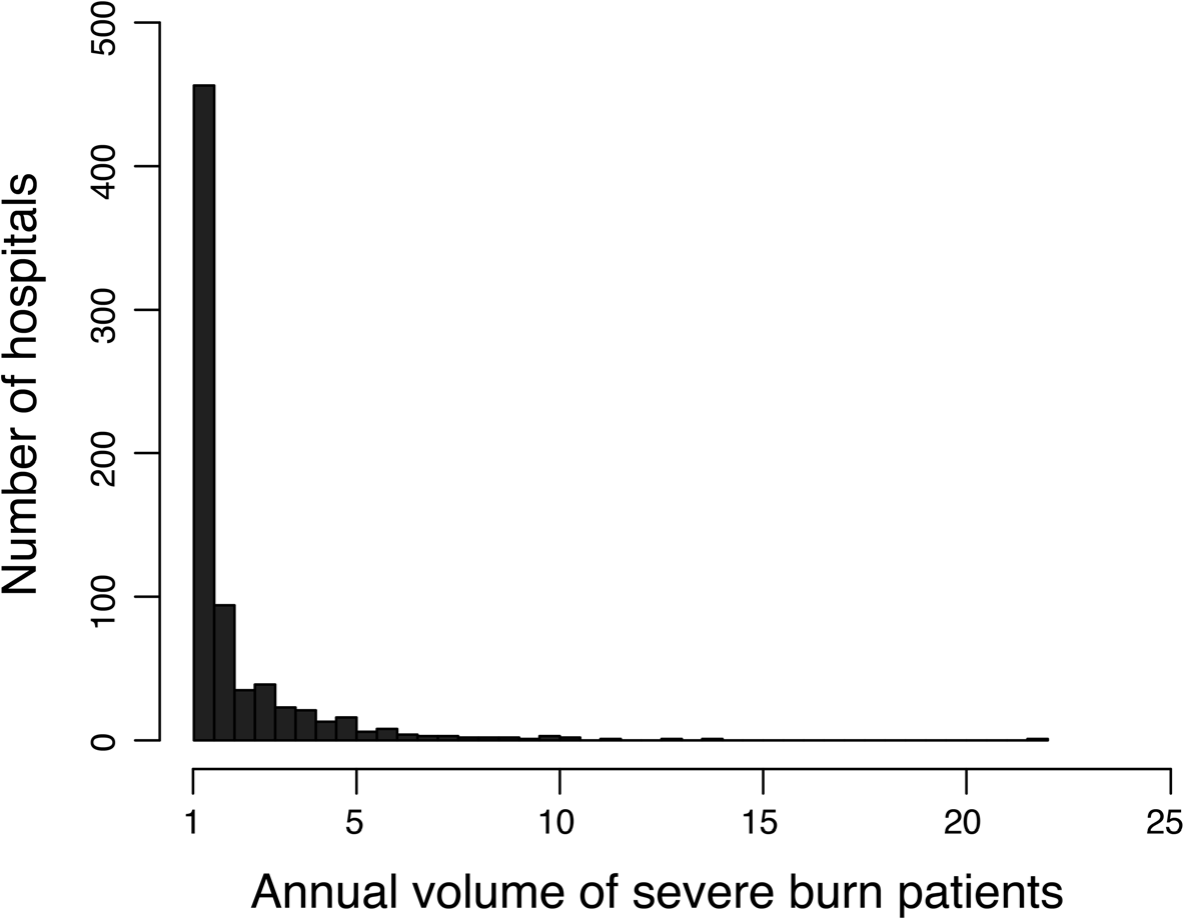
A histogram of hospitals according to their annual severe burn patient volume

Figure 1 shows a histogram of hospital numbers and severe burn patient volumes. A total of 5250 patients from 737 hospitals were eligible for analyses. Approximately 94.6% (697/737) of the analyzed hospitals treated ≤ 5 severe burn patients yearly, and the maximum number of annual burn patients treated per hospital was 22. The characteristics of the multiple imputed cohort dichotomized by annual burn patient volume (≤ 5 or > 5) are presented in Table 1. Patient characteristics in the naïve data, including the proportions of missing data, are presented in Additional file 1: Table S1. Among the entire study cohort, the overall in-hospital mortality rate was 24.5% and median total healthcare costs and hospital-free days at 90 days (interquartile range) were $23,628 (7452, 58,458) and 28 days (0, 67), respectively. There were differences between hospital categories for several variables relating to patient severity; however, PBI had a sufficient discriminative ability with an AUROC of 0.86 in this study cohort **(**Additional file 2: Figure S1A**),** indicating that PBI could be used as a useful risk adjustment measure by itself.

**Table 1:** Patients’ characteristics according to severe burn patient volume (multiple imputed cohort)

Variables	Annual severe burn patients ≤ 5	Annual severe burn patients > 5
Number of hospitals, *n*	697	40
Number of patients, *n*	3648	1602
Transferred from another hospital, *n* (%)	999 (27.4)	539 (33.6)
Year of injury		
2010–2012	1722 (47.2)	756 (47.2)
2013–2015	1926 (52.8)	846 (52.8)
Age, years, median [IQR]	67 [44, 80]	64 [43, 79]
Female sex, *n* (%)	1514 (41.5)	625 (39.0)
Charlson comorbidity index, median [IQR]	0 [0, 1]	0 [0, 0]
Levels of consciousness, alert, *n* (%)	2459 (67.4)	913 (57.0)
Burn index, median [IQR]	15 [10.5, 24.7]	20 [12.5, 35]
Prognostic burn index, median [IQR]	86 [64, 99.5]	90 [68, 106]
Inhalation injury, *n* (%)	560 (15.4)	331 (20.7)
Interventions performed within 2 days of admission		
Intensive care unit, *n* (%)	2033 (55.7)	1367 (85.3)
Mechanical ventilation, *n* (%)	1011 (27.7)	752 (46.9)
Escharotomy, *n* (%)	241 (6.6)	241 (15.0)
Vasopressor, *n* (%)	532 (14.6)	342 (21.3)
Haptoglobin, *n* (%)	267 (7.3)	277 (17.3)
RBC transfusion, *n* (%)	202 (5.5)	130 (8.1)
Skin transplant during hospitalization, *n* (%)	1583 (43.4)	909 (56.7)
Artificial graft use, *n* (%)	314 (8.6)	292 (18.2)
Cultured graft use, *n* (%)	108 (3.0)	136 (8.5)
Hospital characteristics		
A government-approved advanced hospital, *n* (%)	848 (23.2)	732 (45.7)
Number of ICU bed, median [IQR]	3.7 [0, 6.4]	4.9 [3.5, 9.5]
Proportion of transferred patients of a treating hospital, median [IQR]	24.0 [10.5, 39.3]	32.1 [17.2, 46.9]

*IQR* interquartile range, *RBC* red blood cell, *ICU* intensive care unit

**Fig. 2:**
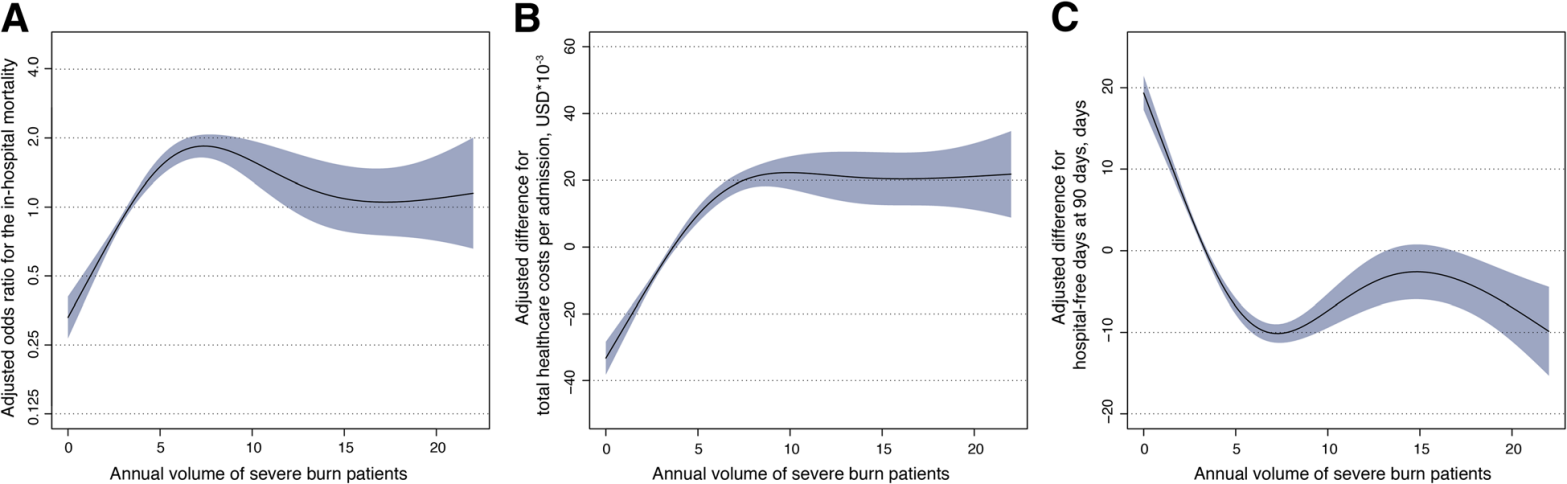
Association between annual severe burn patient volume and adjusted risk of **a** in-hospital survival, **b** total healthcare costs, and **c** hospital-free days at 90 days. The shaded region represents the standard errors for the point estimates. Patient severity was adjusted by the prognostic burn index as a fixed effect variable. The hospital unique identifier was also adjusted as a random effect variable. USD, US dollars

The GAMM plots for in-hospital mortality, total healthcare costs, and hospital-free days at 90 days are shown in Fig. 2. The risk of in-hospital mortality increased up to the patient volume of six per year, and then plateaued, while the total healthcare costs increased according to patient volume up to 10 patients per year, and then plateaued. Moreover, fewer hospital-free days were observed in the higher volume hospitals. In the GLMM analysis in which patient volume was incorporated as a continuous variable, high patient volume was significantly associated with an increased risk of in-hospital mortality (adjusted odds ratio [95% CI] = 1.14 [1.09–1.19] for each patient increase, *p* <  0.001), high total healthcare costs (adjusted difference [95% CI] = $4876 [4436–5316] for each patient increase, *p* <  0.001), and few hospital-free days at 90 days (adjusted difference [95% CI] = − 3.1 days [− 3.4 to − 2.8] for each patient increase, *p* <  0.001) (Table 2). When patient volume was incorporated as a categorical variable into the GLMM analysis, the higher patient volume category was significantly associated with an increased mortality risk (adjusted odds ratio [95% CI] = 1.85 [1.42–2.41], *p* <  0.001), high healthcare costs (adjusted difference [95% CI] = $29,564 [26,237–32,890], *p* <  0.001), and few hospital-free days (adjusted difference [95% CI] = − 15.4 days [− 17.9 to − 13.0], *p* <  0.001) compared to the lower patient volume category (Table 3). These results were consistent with the results of GAMM and GLMM models that used patient volume as a continuous variable.

**Table 2:** Results of the generalized linear mixed model analysis in which patient volume was included as a continuous variable

Population of severe burn patients adjusted by the prognostic burn index					
Primary outcome	*N* (%)	Crude odds ratio (95% CI)	*p* value	Adjusted odds ratio (95% CI)	*p* value
In-hospital mortality	1287 (24.5)	1.07 (1.05–1.09)	< 0.001	1.14 (1.09–1.19)	< 0.001
Secondary outcomes	Median [IQR]	Crude difference (95% CI)	*p* value	Adjusted difference (95% CI)	*p* value
Total healthcare costs per admission, USD	23,628 [7452, 58,458]	3278 (3043–3512)	< 0.001	4876 (4436–5316)	< 0.001
Hospital-free days at 90 days, days	28 [0, 67]	− 1.6 (− 1.7 to − 1.5)	< 0.001	− 3.1 (− 3.4 to − 2.8)	< 0.001
Population who were directly transported from the scene of injury adjusted by the prognostic burn index					
Primary outcome	*N* (%)	Crude odds ratio (95% CI)	*p* value	Adjusted odds ratio (95% CI)	*p* value
In-hospital mortality	1017 (27.3)	1.14 (1.11–1.18)	< 0.001	1.22 (1.17–1.30)	< 0.001
Secondary outcomes	Median [IQR]	Crude difference (95% CI)	*p* value	Adjusted difference (95% CI)	*p* value
Total healthcare costs per admission, USD	22,723 [7087, 58,753]	6191 (5703–6678)	< 0.001	6755 (6076–7434)	< 0.001
Hospital-free days at 90 days, days	22 [0, 66]	− 3.0 (− 3.2 to − 2.7)	< 0.001	− 4.4 (− 4.9 to − 3.9)	< 0.001
Population whose prognostic burn index ≤ 120 adjusted by the prognostic burn index					
Primary outcome	*N* (%)	Crude odds ratio (95% CI)	*p* value	Adjusted odds ratio (95% CI)	*p* value
In-hospital mortality	876 (18.4)	1.07 (1.05–1.09)	< 0.001	1.16 (1.10–1.22)	< 0.001
Secondary outcomes	Median [IQR]	Crude difference (95% CI)	*p* value	Adjusted difference (95% CI)	*p* value
Total healthcare costs per admission, USD	25,289 [8356, 59,223]	3823 (3544–4101)	< 0.001	6021 (5501–6541)	< 0.001
Hospital-free days at 90 days, days	36 [0, 69]	− 1.7 (− 1.9 to − 1.6)	< 0.001	− 3.5 (− 3.8 to − 3.2)	< 0.001
Cohort of severe burn patients who survived for more than 2 days of admission adjusted by the developed risk adjustment model					
Primary outcome	*N* (%)	Crude odds ratio (95% CI)	*p* value	Adjusted odds ratio (95% CI)	*p* value
In-hospital mortality	956 (19.4)	1.07 (1.05–1.09)	< 0.001	1.04 (0.98–1.10)	0.174
Secondary outcomes	Median [IQR]	Crude difference (95% CI)	*p* value	Adjusted difference (95% CI)	*p* value
Total healthcare costs per admission, USD	26,832 [9224, 62,428]	3871 (3604–4138)	< 0.001	6689 (5892–7487)	< 0.001
Hospital-free days at 90 days, days	35 [0, 68]	− 1.7 (− 1.8 to − 1.6)	< 0.001	− 2.1 (− 2.4 to − 1.8)	< 0.001

Patient severity was also adjusted by the hospital unique identifier as a random effect variable. *IQR* interquartile range, *CI* confidence interval, *USD* US dollars

**Table 3:** Results of the generalized linear mixed model analysis in which patient volume was included as a categorical variable (≤ 5 or > 5)

Population of severe burn patients adjusted by the prognostic burn index						
Primary outcome	Hospitals with annual patients ≤ 5, *n* (%)	Hospitals with annual patients > 5, *n* (%)	Crude odds ratio (95% CI)	*p* value	Adjusted odds ratio (95% CI)	*p* value
In-hospital mortality	768 (21.1)	519 (32.4)	1.80 (1.58–2.05)	< 0.001	1.85 (1.42–2.41)	< 0.001
Secondary outcomes	Hospitals with annual patients ≤ 5, median [IQR]	Hospitals with annual patients > 5, median [IQR]	Crude difference (95% CI)	*p* value	Adjusted difference (95% CI)	*p* value
Total healthcare costs per admission, USD	19,500 [6510, 48,618]	35,400 [11,600, 88,034]	27,141 (25,132–29,151)	< 0.001	29,564 (26,237–32,890)	< 0.001
Hospital-free days at 90 days, days	37 [0, 71]	3 [0, 53]	− 12.2 (− 13.2 to − 11.2)	< 0.001	−15.4 (− 17.9 to − 13.0)	< 0.001
Population who were directly transported from the scene of injury adjusted by the prognostic burn index						
Primary outcome	Hospitals with annual patients ≤ 5, *n* (%)	Hospitals with annual patients > 5, *n* (%)	Crude odds ratio (95% CI)	*p* value	Adjusted odds ratio (95% CI)	*p* value
In-hospital mortality	792 (26.1)	225 (33.1)	1.40 (1.17–1.68)	< 0.001	1.40 (0.97–2.02)	0.072
Secondary outcomes	Hospitals with annual patients ≤ 5, median [IQR]	Hospitals with annual patients > 5, median [IQR]	Crude difference (95% CI)	*p* value	Adjusted difference (95% CI)	*p* value
Total healthcare costs per admission, USD	20,657 [6645, 54,832]	32,934 [9553, 83,600]	23,169 (20,369–25,969)	< 0.001	24,677 (20,236–29,119)	< 0.001
Hospital-free days at 90 days, days	27 [0, 68]	0 [0, 54]	− 8.5 (− 9.9 to − 7.1)	< 0.001	− 11.8 (− 15.3 to − 8.3)	< 0.001
Population whose prognostic burn index ≥ 120 adjusted by the prognostic burn index						
Primary outcome	Hospitals with annual patients ≤ 5, *n* (%)	Hospitals with annual patients > 5, *n* (%)	Crude odds ratio (95% CI)	*p* value	Adjusted odds ratio (95% CI)	*p* value
In-hospital mortality	600 (16.6)	276 (23.9)	1.57 (1.34–1.85)	< 0.001	1.77 (1.31–2.38)	< 0.001
Secondary outcomes	Hospitals with annual patients ≤ 5, median [IQR]	Hospitals with annual patients > 5, median [IQR]	Crude difference (95% CI)	*p* value	Adjusted difference (95% CI)	*p* value
Total healthcare costs per admission, USD	21,565 [7193, 52,228]	37,724 [15,388, 87,400]	25,201 (22,995–27,408)	< 0.001	29,976 (26,068–33,885)	< 0.001
Hospital-free days at 90 days, days	41 [0, 71]	19 [0, 57.25]	− 10.5 (− 11.6 to − 9.4)	< 0.001	− 15.3 (− 18.0 to − 12.6)	< 0.001
Cohort of severe burn patients who survived for more than 2 days of admission adjusted by the developed risk adjustment model						
Primary outcome	Hospitals with annual patients ≤ 5, *n* (%)	Hospitals with annual patients > 5, *n* (%)	Crude odds ratio (95% CI)	*p* value	Adjusted odds ratio (95% CI)	*p* value
In-hospital mortality	615 (17.1)	341 (25.6)	1.66 (1.43–1.93)	< 0.001	0.97 (0.71–1.33)	0.853
Secondary outcomes	Hospitals with annual patients ≤ 5, median [IQR]	Hospitals with annual patients > 5, median [IQR]	Crude difference (95% CI)	*p* value	Adjusted difference (95% CI)	*p* value
Total healthcare costs per admission, USD	22,516 [7668, 53,255]	39,157 [16,339, 94,715]	27,999 (25,805–30,192)	< 0.001	22,750 (16,969–28,530)	< 0.001
Hospital-free days at 90 days, days	40 [0, 71]	17.5 [0, 57.25]	−10.6 (−11.6 to − 9.6)	< 0.001	− 7.3 (− 8.7 to − 5.8)	< 0.001

Patient severity was also adjusted by the hospital unique identifier as a random effect variable. *IQR* interquartile range, *CI* confidence interval, *USD* US dollars

The quantitative bias analysis showed that the estimated *E* value was 1.34 in the main analysis, meaning that the observed association with adjusted odds ratio of 1.14 could be explained away by a possible unmeasured confounder that was associated with both the patient volume and the in-hospital mortality by an odds ratio of 1.34-fold each, on the condition with the adjustment for PBI, but weaker confounding could not do so. Also, the estimated null *E* value was 1.26, meaning that the confidence interval could be moved to include the null by a possible unmeasured confounder, that was associated with both the patient volume and in-hospital mortality, by an odds ratio of 1.26-fold each, on the condition with the adjustment for PBI.

### Sensitivity analyses

A histogram of the hospital numbers and the volume of severe burn patients who were directly transported from the scene of injury is shown in Additional file 3: Figure S2. A total of 3712 patients from 625 hospitals were analyzed. Patient characteristics of the multiple imputed and the naïve data are presented in Additional file 4: Table S2 and Additional file 5: Table S3, respectively. PBI had sufficient discriminative ability with an AUROC of 0.86 (Additional file 2: Figure S1A). The GAMM plots of this cohort showed similar trends compared to those of the original study population (Additional file 6: Figure S3). In the GLMM model, in which patient volume was incorporated as a continuous variable, high patient volume was significantly associated with increased risk of in-hospital mortality (adjusted odds ratio [95% CI] = 1.22 [1.17–1.30] for each patient increase, *p* <  0.001), high total healthcare costs (adjusted difference [95% CI] = $6755 [6076–7434] for each patient increase, *p* <  0.001), and few hospital-free days at 90 days (adjusted difference [95% CI] = − 4.4 days [− 4.9 to − 3.9] for each patient increase, *p* <  0.001) (Table 2). When patient volume was incorporated as a categorical variable into the GLMM model, although a high patient volume was significantly associated with high total healthcare costs and few hospital-free days at 90 days, the difference was not significant for in-hospital mortality (adjusted odds ratio [95% CI] = 1.40 [0.97–2.07] for each patient increase, *p* = 0.072) (Table 3).

A histogram of the hospital numbers and the volume of severe burn patients with PBI ≤ 120 is shown in Additional file 7: Figure S4. A total of 4766 patients from 729 hospitals were analyzed. Patient characteristics of the multiple imputed and the naïve data are presented in Additional file 8: Table S4 and Additional file 9: Table S5, respectively. The AUROC of PBI for predicting in-hospital mortality was 0.82 in this population (Additional file 2: Figure S1A). The GAMM plots of this cohort showed similar trends compared to those of the original study population (Additional file 10: Figure S5). In the GLMM model, in which patient volume was incorporated as a continuous variable, high patient volume was significantly associated with increased risk of in-hospital mortality (adjusted odds ratio [95% CI] = 1.16 [1.10–1.22] for each patient increase, *p* <  0.001), high total healthcare costs (adjusted difference [95% CI] = $6021 [5501–6541] for each patient increase, *p* <  0.001), and few hospital-free days at 90 days (adjusted difference [95% CI] = − 3.5 days [− 3.8 to − 3.2] for each patient increase, *p* <  0.001) (Table 2). When patient volume was incorporated as a categorical variable into the GLMM model, a high patient volume was significantly associated with increased risk of in-hospital mortality, high total healthcare costs, and few hospital-free days at 90 days (Table 3).

For the population excluding patients who died within 2 days of admission, the risk adjustment model that we developed in this cohort had higher discriminative performance than PBI with an AUROC of 0.89 in the establishment cohort. The risk adjustment model was well calibrated in the validation cohort with an AUROC of 0.89, and the Hosmer-Lemeshow goodness-of-fit test had a *p* value of 0.425 (Additional file 2: Figure S1A and S1B). The issue of multicollinearity was eliminated in the regression model for risk adjustment since all the variance inflation factors in each variable were lower than two. A histogram of the hospital numbers and the volume of severe burn patients who survived for more than 2 days of admission is shown in Additional file 11: Figure S6. A total of 4919 patients from 736 hospitals were analyzed. Patient characteristics of the multiple imputed and the naïve data are presented in Additional file 12: Table S6 and Additional file 13: Table S7, respectively. The GAMM plots for all of the outcomes showed similar trends compared to those of the original study cohort (Additional file 14: Figure S7). In the GLMM model in which patient volume was incorporated as a continuous variable, although high patient volume was significantly associated with high total healthcare costs and few hospital-free days at 90 days, statistically significant difference was not observed for in-hospital mortality (adjusted odds ratio [95% CI] = 1.04 [0.98–1.10] for each patient increase, *p* = 0.174) (Table 2). The findings from the GLMM model, in which patient volume was incorporated as a categorical variable, were similar to those in which patient volume was incorporated as a continuous variable (Table 3).

Results of the cost analysis with additional adjustment for the length of hospital stay using GAMM and GLMM models were similar compared to those without adjustment for the length of hospital stay (Additional file 15: Figure S8 and Additional file 16: Table S8).

## Discussion

In the present study, we retrospectively analyzed the Japanese nationwide administrative database and evaluated the association between patient volume per hospital and the outcomes in 5250 severe burn patients. To the best of our knowledge, this study was the first to demonstrate the association between severe burn patient volume and healthcare costs. Our findings demonstrated that a high severe burn patient volume was significantly associated with increased in-hospital mortality, high total healthcare costs per admission, and few hospital-free days, at least in current Japanese settings. As the DPC database contained almost all of the tertiary emergency medical centers and the majority of emergency hospitals, it could be assumed to represent real-world data in Japan. Several previous studies that have analyzed the DPC database showed the positive volume-outcome relationship for various diseases and procedures, such as severe trauma [3], severe acute pancreatitis [27], stroke [28], pancreaticoduodenectomy [29], and liver resection [30]. However, in this study, the favorable effects according to patient volume were not observed in severe burn patients, suggesting the necessity of further improvements in the Japanese severe burn care system from the perspective of patient mortality and socioeconomic costs.

As specialized management is generally required in severe burn patients, a volume-outcome relationship is theoretically expected in those patients. Recent reviews and practice guidelines recommended centralizing burn patients to specialized hospitals that receive a sufficient number of patients [6, 31], and several countries, such as the USA and the UK, have established specific burn centers and have employed a patient centralizing system based on this assumption. However, there has been insufficient evidence justifying this strategy. Hranjec et al. [9] showed that hospitals with high burn volumes had the highest risk of mortality, and Pacella et al. [10] demonstrated that the mortality rate in high-volume hospitals was significantly higher than those in medium-low-volume hospitals in patients with extensive third-degree burns. Although another study [8] showed a volume-dependent decrease in mortality, it only included a pediatric population. Light et al. [32] reported that mortality did not linearly improve with patient volume; they found that it plateaued with increasing patient volumes. Furthermore, it should be noted that most of those studies included less severe cases that did not require specialized strategies.

On the other hand, it was reported that the treating hospital was an independent prognostic factor, in addition to already known risk factors such as age and the percentage of total body surface area burnt [9, 32]. It was also reported that there were significant differences in clinical practice between hospitals even though they were burn centers [33]. A previous study [34] reported that the magnitude of specialization, defined as caseload volume for the disease investigated to the overall throughput of the hospital, would affect patient outcomes in addition to conventional caseload volume measures. Well-performing hospitals might manage patients using specialized assets in addition to experience (patient volume), which could not be evaluated in database analyses. As Japan is lacking a centralized system for severe burn patients, severe burn patients are usually transferred to regional tertiary emergency hospitals where burn experts are not always available. Hence, excessive admission of severe burn patients could overwhelm the hospitals’ capacity due to a shortage of materials and human resources. This could be one explanation for our findings that demonstrated high mortality rates in high volume facilities. A positive volume-outcome relationship might be achieved by the adequate centralization of not only patients but also healthcare resources.

In the present study, high severe burn patient volume was significantly associated with high total healthcare costs after adjustments for patient severity and length of hospital stay. As total healthcare costs evaluated in this study included some kinds of additional claims approved in only specific burn treating hospitals, high healthcare costs did not directly reflect the high intensity of treatments. However, our findings suggested that high volume hospitals provided a higher intensity of care during hospitalization. Specialized care for severe burn patients generally requires a multidisciplinary approach such as aggressive fluid resuscitation, analgesia, multistage escharotomy and skin grafting, and mental care [35–37]. In this study, high-intensity procedures such as mechanical ventilation, early phase escharotomy, or skin transplants were more frequently provided in high volume hospitals. Furthermore, our data also showed that high volume facilities were more likely to use expensive artificial or autologous cultured skin grafts. Although these differences could be partially explained by the differences in patient severity, differences in standard operating procedures between facilities (i.e., hospital preferences) would affect the daily practices and thus raise total healthcare costs. With the recent improvement in the mortality of severe burn patients, it has been reported that overall mortality was insufficient as an outcome measure and the importance of functional, scar, and psychological outcomes has been raised as clinical practice indicators [6, 38]. However, in this study, cost analyses for each practice and unrecorded outcomes such as mental, pain, and scar condition could not be conducted. Further large-scale studies investigating such indicators are necessary in the future.

Several limitations should be considered in interpreting the results of this study. First, this was a retrospective study that used an administrative database; therefore, unmeasured variables (e.g., vital signs, laboratory data, and burn mechanism: scald, flame, or chemical) that could influence the outcomes were not accounted for. Second, the smaller severe burn patient volume per hospital in Japan prevented the analysis of volume-outcome relationship for a larger range of patient volumes. Furthermore, because the number of high volume hospitals was limited, the outcomes in these high-volume hospitals would affect the results excessively. Third, the Japanese healthcare insurance system is generally not applied for specific cases, such as labor-related accidents, and the DPC database did not contain such cases. Fourth, we lacked data about the detailed quality and processes involved in multidisciplinary burn care that may contribute to the causal pathway linking hospital burn patient volumes and outcomes. Finally, because our results reflected only the settings of a country, further studies are needed to validate whether our results are applicable globally. Despite these limitations, this was the first large-scale observational study using real-world data that evaluated the current Japanese situation regarding severe burn care. Our findings demonstrated a significant association between high patient volumes and unfavorable outcomes, suggesting that experience (patient volume) only would be insufficient to achieve theoretically expected positive volume-outcome relationship in severe burn care, from the perspective of patient mortality and socioeconomic costs. A detailed review of the current Japanese system is necessary to identify the points for future improvements in severe burn care.

## Conclusions

The analysis of the Japanese nationwide administrative database study demonstrated that high volumes of severe burn patients were significantly associated with increased in-hospital mortality, high healthcare costs, and long duration of hospital stay. Because of the lack of generalizability and the shortage of high-volume hospitals, further studies are needed to validate our results.

## Additional files

Additional file 1: **Table S1.** Patient characteristics of the entire study population (naïve data). (DOCX 19 kb)

Additional file 2: **Figure S1.** A, Receiver operating curves of the prognostic burn index and the developed risk adjustment model; B, Calibration plot. AUROC, area under the receiver operating curve. (TIF 985 kb)

Additional file 3: **Figure S2.** Histogram of the hospital numbers and the volume of severe burn patients who were directly transported from the scene of injury. (TIF 1230 kb)

Additional file 4: **Table S2.** Characteristics of the severe burn patients who were directly transported from the scene of injury (multiple imputed data). (DOCX 18 kb)

Additional file 5: **Table S3.** Characteristics of the severe burn patients who were directly transported from the scene of injury (naïve data). (DOCX 19 kb)

Additional file 6: **Figure S3.** Association between annual severe burn patient volume and adjusted risk of (A) in-hospital survival, (B) total healthcare costs, and (C) hospital-free days at 90 days among patients who were directly transported from the scene of injury. The shaded region represents the standard errors for the point estimates. Patient severity was adjusted by prognostic burn index as a fixed effect variable. The hospital unique identifier was also adjusted as a random effect variable. Abbreviation: USD, US dollars. (TIF 1342 kb)

Additional file 7: **Figure S4.** Histogram of the hospital numbers and the volume of severe burn patients with prognostic burn index ≤ 120. (TIF 493 kb)

Additional file 8: **Table S4.** Characteristics of the severe burn patients with prognostic burn index ≤ 120 (multiple imputed data). (DOCX 19 kb)

Additional file 9: **Table S5.** Characteristics of the severe burn patients who survived for patients with prognostic burn index ≤ 120 (naïve data). (DOCX 19 kb)

Additional file 10: **Figure S5.** Association between annual severe burn patient volume and adjusted risk of (A) in-hospital survival, (B) total healthcare costs, and (C) hospital-free days at 90 days among patients with prognostic burn index ≤ 120. The shaded region represents the standard errors for the point estimates. Patient severity was adjusted by prognostic burn index as a fixed effect variable. The hospital unique identifier was also adjusted as a random effect variable. Abbreviation: USD, US dollars. (TIF 1331 kb)

Additional file 11: **Figure S6.** Histogram of the hospital numbers and the volume of severe burn patients who survived for more than 2 days of admission. (TIF 1103 kb)

Additional file 12: **Table S6.** Characteristics of the severe burn patients who survived for more than 2 days of admission (multiple imputed data) (DOCX 19 kb)

Additional file 13: **Table S7.** Characteristics of the severe burn patients who survived for patients who survived for more than 2 days of admission (naïve data). (DOCX 19 kb)

Additional file 14: **Figure S7.** Association between annual severe burn patient volume and the adjusted risk of (A) in-hospital survival, (B) total healthcare costs, and (C) hospital-free days at 90 days among patients who survived for more than 2 days of admission. The shaded region represents the standard errors for the point estimates. Patient severity was adjusted by the developed risk adjustment model as a fixed effect variable. The hospital unique identifier was also adjusted as a random effect variable. Abbreviation: USD, US dollars. (TIF 1282 kb)

Additional file 15: **Figure S8.** Association between the annual severe burn patient volume and the adjusted difference of total healthcare costs with additional adjustment for the length of hospital stay in the (A) entire study population, (B) population who were directly transported from the scene of injury, (C) population with prognostic burn index ≤ 120, and (D) population excluding patients who died within 2 days of admission. The shaded region represents the standard errors for the point estimates. Patient severity was adjusted by the prognostic burn index in (A), (B), and (C) and by the developed risk adjustment model in (D). The hospital unique identifier was also adjusted as a random effect variable. Abbreviation: USD, US dollars. (TIF 1590 kb)

Additional file 16: **Table S8**. Results of the generalized linear mixed-effect model analysis for total healthcare costs with additional adjustment for the length of hospital stay. (DOCX 15 kb)

## Abbreviations

AUROC: Area under the receiver operating curve
CI: Confidence interval
DPC: Diagnosis procedure combination
GAMM: Generalized additive mixed-effect model
GLMM: Generalized linear mixed model
ICD-10: International Classification of Diseases, 10th Revision
ICU: Intensive care unit
IQR: Interquartile range
RBC: Red blood cell

## References

[1] Smolle C, Cambiaso-Daniel J, Forbes AA, Wurzer P, Hundeshagen G, Branski LK (2017). Recent trends in burn epidemiology worldwide: a systematic review. Burns.

[2] Forjuoh SN (2006). Burns in low- and middle-income countries: a review of available literature on descriptive epidemiology, risk factors, treatment, and prevention. Burns.

[3] Endo A, Shiraishi A, Fushimi K, Murata K, Otomo Y. Increased severe trauma patient volume is associated with survival benefit and reduced total health care costs: a retrospective observational study using a Japanese Nationwide Administrative Database. Ann Surg. 2017. 10.1097/SLA.0000000000002324. 10.1097/SLA.0000000000002324 28594743

[4] Schrag D, Cramer LD, Bach PB, Cohen AM, Warren JL, Begg CB (2000). Influence of hospital procedure volume on outcomes following surgery for colon cancer. JAMA.

[5] Hata T, Motoi F, Ishida M, Naitoh T, Katayose Y, Egawa S (2016). Effect of hospital volume on surgical outcomes after pancreaticoduodenectomy: a systematic review and meta-analysis. Ann Surg.

[6] Hardwicke J (2016). The influence of outcomes on the provision and practice of burn care. Burns.

[7] Hodgman EI, Saeman MR, Subramanian M, Wolf SE (2016). The effect of burn center volume on mortality in a pediatric population: an analysis of the National Burn Repository. J Burn Care Res..

[8] Palmieri TL, Taylor S, Lawless M, Curri T, Sen S, Greenhalgh DG (2015). Burn center volume makes a difference for burned children. Pediatr Crit Care Med.

[9] Hranjec T, Turrentine FE, Stukenborg G, Young JS, Sawyer RG, Calland JF (2012). Burn-center quality improvement: are burn outcomes dependent on admitting facilities and is there a volume-outcome "sweet-spot"?. Am Surg.

[10] Pacella SJ, Butz DA, Comstock MC, Harkins DR, Kuzon WM, Taheri PA (2006). Hospital volume outcome and discharge disposition of burn patients. Plast Reconstr Surg.

[11] Osler T, Glance LG, Hosmer DW (2011). Comparison of hospital mortality rates after burn injury in New York state: a risk-adjusted population-based observational study. J Trauma.

[12] Yasunaga H, Matsui H, Horiguchi H, Fushimi K, Matsuda S (2013). Clinical epidemiology and health services research using the Diagnosis Procedure Combination Database in Japan. Asian Pac J Dis Manage.

[13] Nakae H, Wada H. Characteristics of burn patients transported by ambulance to treatment facilities in AkitaPrefecture, Japan. Burns. 2002;28:73–9. 10.1016/s0305-4179(01)00063-8 11834335

[14] Charlson ME, Pompei P, Ales KL, MacKenzie CR (1987). A new method of classifying prognostic comorbidity in longitudinal studies: development and validation. J Chronic Dis.

[15] Quan H, Sundararajan V, Halfon P, Fong A, Burnand B, Luthi JC (2005). Coding algorithms for defining comorbidities in ICD-9-CM and ICD-10 administrative data. Med Care.

[16] Yasuda K, Henmi H, Yamamoto Y, Mashiko K, Otomo Y, Ohtsuka T (1986). Nutritional management and assessment on extensively burned patients (in Japanese). Jpn J Burn Inj.

[17] Sheppard NN, Hemington-Gorse S, Shelley OP, Philp B, Dziewulski P (2011). Prognostic scoring systems in burns: a review. Burns.

[18] Morita S, Higami S, Yamagiwa T, Iizuka S, Nakagawa Y, Yamamoto I (2010). Characteristics of elderly Japanese patients with severe burns. Burns.

[19] Tagami T, Matsui H, Fushimi K, Yasunaga H (2015). Validation of the prognostic burn index: a nationwide retrospective study. Burns.

[20] Young P, Hodgson C, Dulhunty J, Saxena M, Bailey M, Bellomo R (2012). End points for phase II trials in intensive care: recommendations from the Australian and New Zealand Clinical Trials Group consensus panel meeting. Crit Care Resusc.

[21] Rubin DB (1987). Multiple imputation for nonresponse in surveys.

[22] Wood SN (2011). Fast stable restricted maximum likelihood and marginal likelihood estimation of semiparametric generalized linear models. J R Stat Soc Series B Stat Methodol.

[23] Tagami T, Matsui H, Fushimi K, Yasunaga H (2016). Prophylactic antibiotics may improve outcome in patients with severe burns requiring mechanical ventilation: propensity score analysis of a Japanese Nationwide Database. Clin Infect Dis.

[24] Tagami T, Matsui H, Moroe Y, Fukuda R, Shibata A, Tanaka C (2017). Antithrombin use and 28-day in-hospital mortality among severe-burn patients: an observational nationwide study. Ann Intensive Care.

[25] VanderWeele TJ, Ding P (2017). Sensitivity analysis in observational research: introducing the E-value. Ann Intern Med.

[26] Ding P, VanderWeele TJ (2016). Sensitivity analysis without assumptions. Epidemiology.

[27] Murata A, Matsuda S, Mayumi T, Yokoe M, Kuwabara K, Ichimiya Y (2011). Effect of hospital volume on clinical outcome in patients with acute pancreatitis, based on a national administrative database. Pancreas.

[28] Tsugawa Y, Kumamaru H, Yasunaga H, Hashimoto H, Horiguchi H, Ayanian JZ (2013). The association of hospital volume with mortality and costs of care for stroke in Japan. Med Care.

[29] Yoshioka R, Yasunaga H, Hasegawa K, Horiguchi H, Fushimi K, Aoki T (2014). Impact of hospital volume on hospital mortality, length of stay and total costs after pancreaticoduodenectomy. Br J Surg.

[30] Yasunaga H, Horiguchi H, Matsuda S, Fushimi K, Hashimoto H, Ohe K (2012). Relationship between hospital volume and operative mortality for liver resection: data from the Japanese Diagnosis Procedure Combination database. Hepatol Res.

[31] ISBI Practice Guidelines Committee (2016). ISBI practice guidelines for burn care. Burns.

[32] Light TD, Latenser BA, Kealey GP, Wibbenmeyer LA, Rosenthal GE, Sarrazin MV (2009). The effect of burn center and burn center volume on the mortality of burned adults--an analysis of the data in the National Burn Repository. J Burn Care Res..

[33] Garside TL, Lee RP, Delaney A, Milliss D (2018). Clinical practice variation in acute severe burn injury. Anaesth Intensive Care.

[34] Lee KC, Sethuraman K, Yong J (2015). On the hospital volume and outcome relationship: does specialization matter more than volume?. Health Serv Res.

[35] Henschke A, Lee R, Delaney A (2016). Burns management in ICU: quality of the evidence: a systematic review. Burns.

[36] Al-Mousawi AM, Mecott-Rivera GA, Jeschke MG, Herndon DN (2009). Burn teams and burn centers: the importance of a comprehensive team approach to burn care. Clin Plast Surg.

[37] Orgill DP, Piccolo N (2009). Escharotomy and decompressive therapies in burns. J Burn Care Res.

[38] Pereira C, Murphy K, Herndon D (2004). Outcome measures in burn care. Is mortality dead?. Burns.

