# Supplementary material for: Volume-outcome relationship on survival and cost benefits in severe burn injury: a retrospective analysis of a Japanese nationwide administrative database
Source: J Intensive Care. 2019 Jan 30;7:7. doi: 10.1186/s40560-019-0363-7 (PMC6354429; doi:10.1186/s40560-019-0363-7)
Supplement: Supplementary file 1 — Table S1. Patient characteristics of the entire study population (naïve data). (DOCX 19 kb) [file 40560_2019_363_MOESM1_ESM.docx]

| **Supplementary Table 1. Patient characteristics of the entire study population (naïve data)** | | | | | |
| --- | --- | --- | --- | --- | --- |
| Variables | | Annual severe burn patients ≤5 | | Annual severe burn patients >5 | |
|  |  | Registered | Missing, n (%) | Registered | Missing, n (%) |
| Number of hospitals, n | | 697 | 0 (0) | 40 | 0 (0) |
| Number of patients, n | | 3648 | 0 (0) | 1602 | 0 (0) |
| Transferred from another hospital, n (%) | | 999 (27.4) | 0 (0) | 539 (33.6) | 0 (0) |
| Year of injury | |  | 0 (0) |  | 0 (0) |
|  | 2010–2012 | 1722 (47.2) | - | 756 (47.2) | - |
|  | 2013–2015 | 1926 (52.8) | - | 846 (52.8) | - |
| Age, years, median [IQR] | | 67 [44, 80] | 0 (0) | 64 [43, 79] | 0 (0) |
| Female sex, n (%) | | 1514 (41.5) | 0 (0) | 625 (39.0) | 0 (0) |
| Charlson comorbidity index, median (IQR) | | 0 [0, 1] | 0 (0) | 0 [0, 0] | 0 (0) |
| Levels of consciousness, alert, n (%) | | 2459 (67.4) | 0 (0) | 913 (57.0) | 0 (0) |
| Burn index, median (IQR) | | 15 [10.5, 24.7] | 0 (0) | 20 [12.5, 35] | 0 (0) |
| Prognostic burn index, median (IQR) | | 86 [64, 99.5] | 0 (0) | 90 [68, 106] | 0 (0) |
| Inhalation injury, n (%) | | 560 (15.4) | 0 (0) | 331 (20.7) | 0 (0) |
| Interventions performed within 2 days of admission | | | | | |
|  | Intensive care unit, n (%) | 1996 (54.7) | 244 (6.7) | 1354 (84.5) | 48 (3.0) |
|  | Mechanical ventilation, n (%) | 2398 (65.7) | 244 (6.7) | 805 (50.2) | 48 (3.0) |
|  | Escharotomy, n (%) | 237 (6.5) | 244 (6.7) | 240 (15.0) | 48 (3.0) |
|  | Vasopressor, n (%) | 529 (14.5) | 244 (6.7) | 341 (21.3) | 48 (3.0) |
|  | Haptoglobin, n (%) | 266 (7.3) | 244 (6.7) | 276 (17.2) | 48 (3.0) |
|  | RBC transfusion, n (%) | 199 (5.5) | 244 (6.7) | 129 (8.1) | 48 (3.0) |
| Skin transplant during hospitalization, n (%) | | 1543 (42.3) | 244 (6.7) | 901 (56.2) | 48 (3.0) |
|  | Artificial graft use, n (%) | 311 (8.5) | 244 (6.7) | 291 (18.2) | 48 (3.0) |
|  | Cultured graft use, n (%) | 107 (2.9) | 244 (6.7) | 136 (8.5) | 48 (3.0) |
| Hospital characteristics | | | | | |
|  | A government-approved advanced hospital, n (%) | 848 (23.2) | 0 (0) | 732 (45.7) | 0 (0) |
|  | Number of ICU bed, median (IQR) | 3.7 [0, 6.4] | 0 (0) | 4.9 [3.5, 9.5] | 0 (0) |
|  | Proportion of transferred patients of a treating hospital, median (IQR) | 24.0 [10.5, 39.3] | 0 (0) | 32.1 [17.2, 46.9] | 0 (0) |
| Abbreviation: IQR, interquartile range; RBC, red blood cell; ICU, intensive care unit | | | | | |
