# Supplementary figures and images for: Volume-outcome relationship on survival and cost benefits in severe burn injury: a retrospective analysis of a Japanese nationwide administrative database
Source: J Intensive Care. 2019 Jan 30;7:7. doi: 10.1186/s40560-019-0363-7 (PMC6354429; doi:10.1186/s40560-019-0363-7)

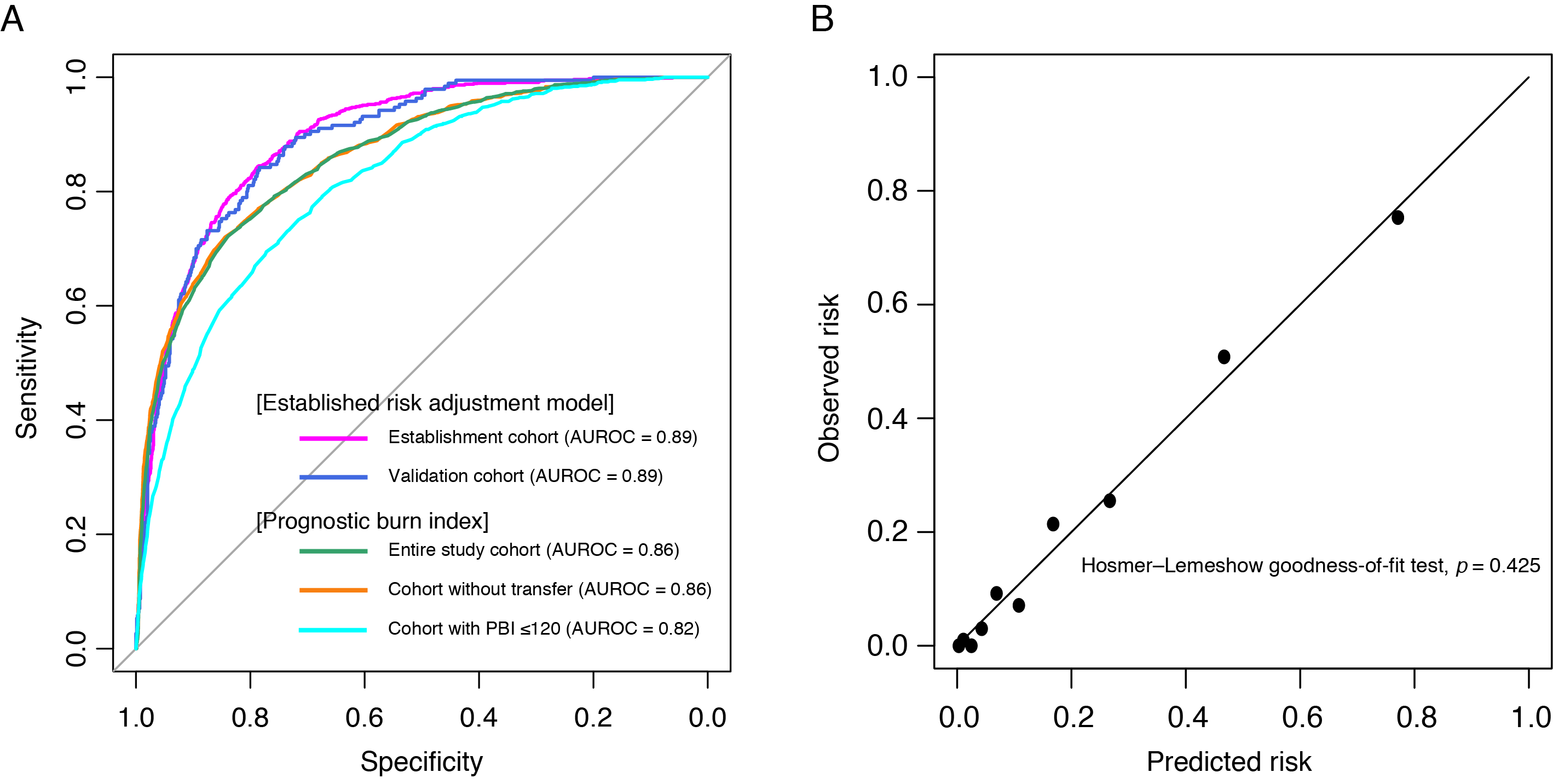

Supplement: Supplementary file 2 — Figure S1. A, Receiver operating curves of the prognostic burn index and the developed risk adjustment model; B, Calibration plot. AUROC, area under the receiver operating curve. (TIF 985 kb) [file 40560_2019_363_MOESM2_ESM.tif]

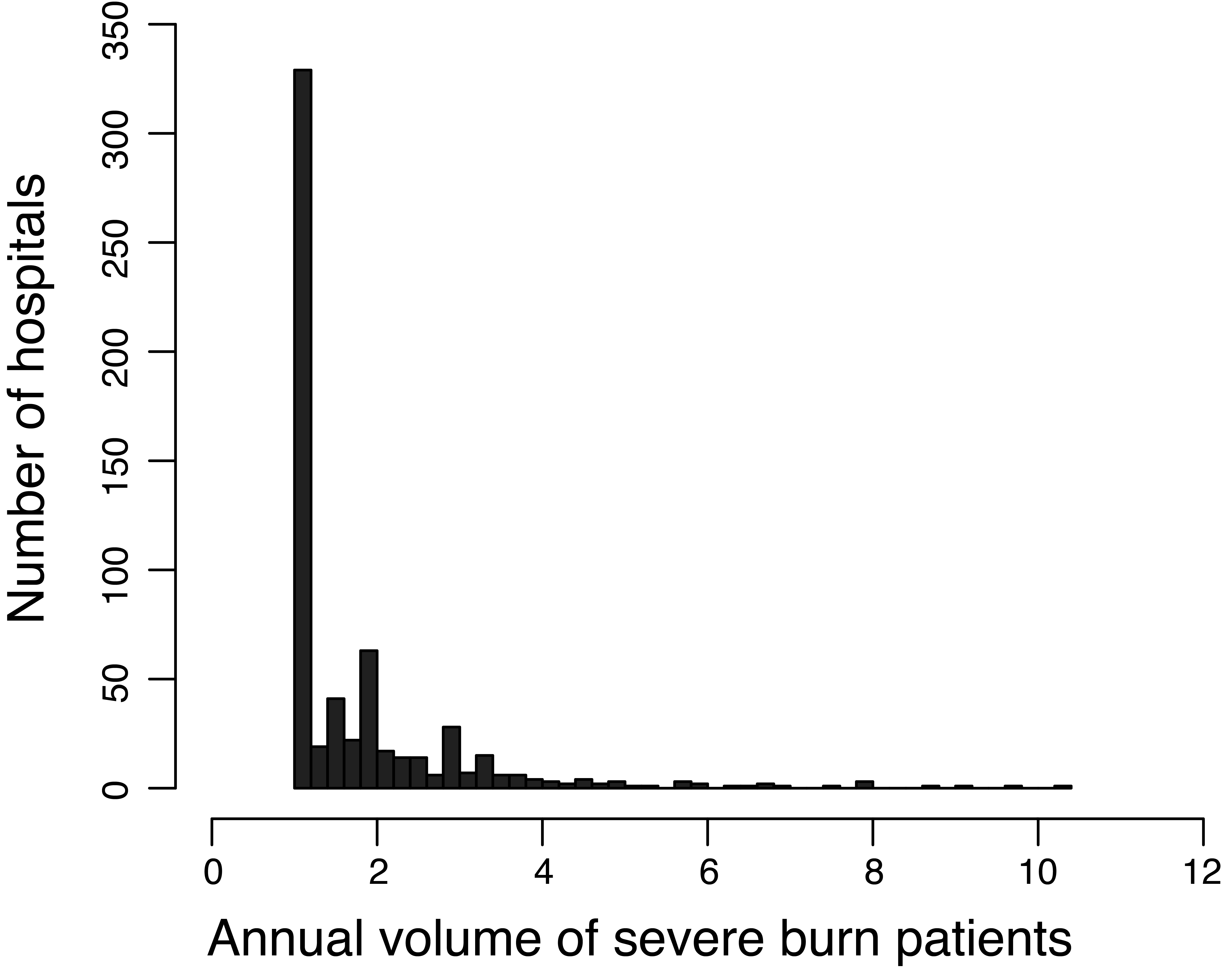

Supplement: Supplementary file 3 — Figure S2. Histogram of the hospital numbers and the volume of severe burn patients who were directly transported from the scene of injury. (TIF 1230 kb) [file 40560_2019_363_MOESM3_ESM.tif]

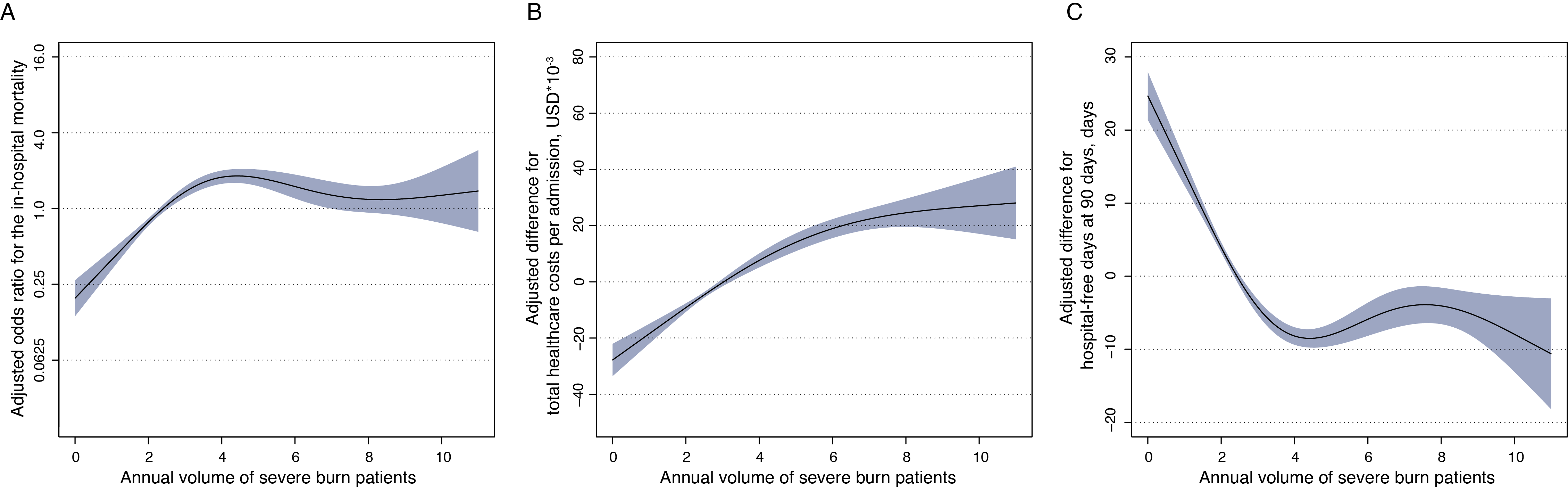

Supplement: Supplementary file 6 — Figure S3. Association between annual severe burn patient volume and adjusted risk of (A) in-hospital survival, (B) total healthcare costs, and (C) hospital-free days at 90 days among patients who were directly transported from the scene of injury. The shaded region represents the standard errors for the point estimates. Patient severity was adjusted by prognostic burn index as a fixed effect variable. The hospital unique identifier was also adjusted as a random effect variable. Abbreviation: USD, US dollars. (TIF 1342 kb) [file 40560_2019_363_MOESM6_ESM.tif]

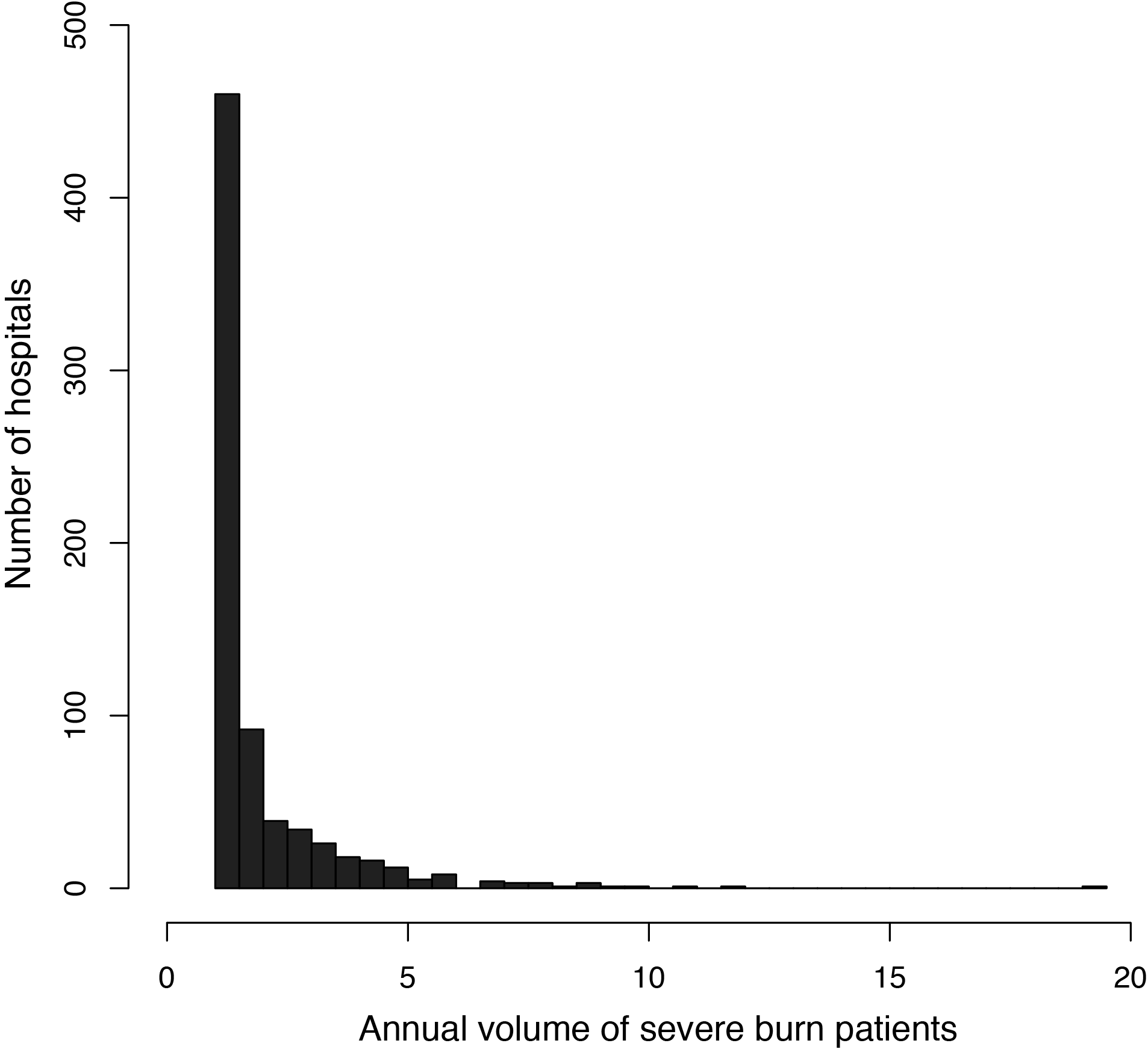

Supplement: Supplementary file 7 — Figure S4. Histogram of the hospital numbers and the volume of severe burn patients with prognostic burn index ≤ 120. (TIF 493 kb) [file 40560_2019_363_MOESM7_ESM.tif]

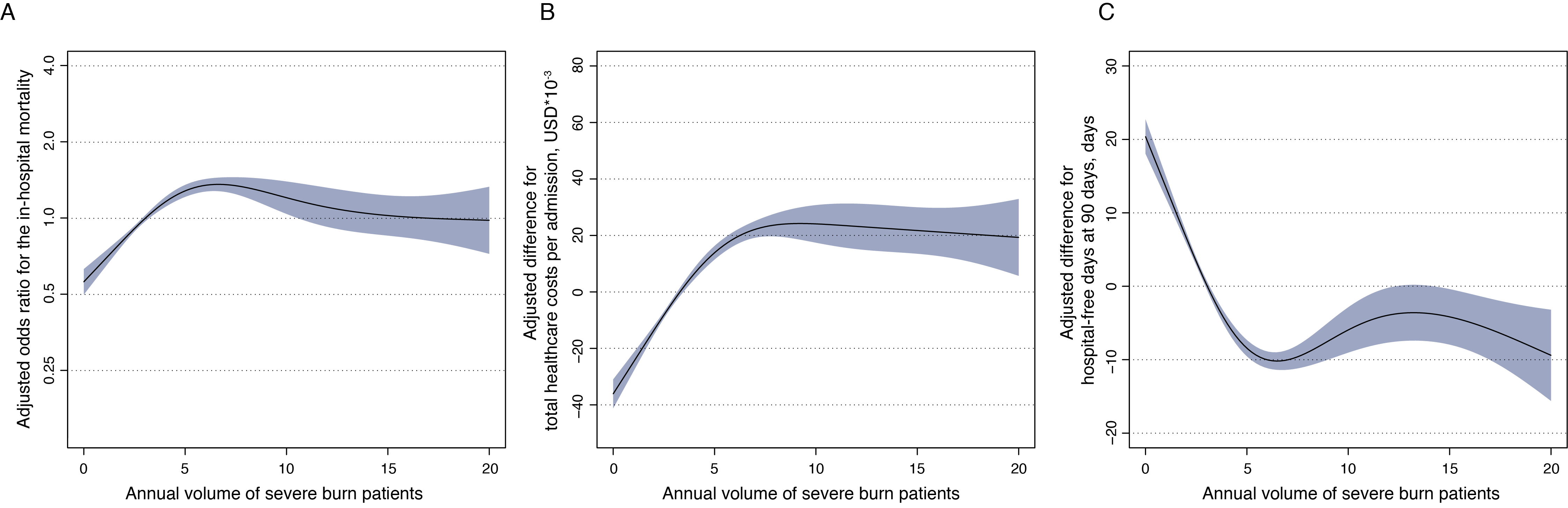

Supplement: Supplementary file 10 — Figure S5. Association between annual severe burn patient volume and adjusted risk of (A) in-hospital survival, (B) total healthcare costs, and (C) hospital-free days at 90 days among patients with prognostic burn index ≤ 120. The shaded region represents the standard errors for the point estimates. Patient severity was adjusted by prognostic burn index as a fixed effect variable. The hospital unique identifier was also adjusted as a random effect variable. Abbreviation: USD, US dollars. (TIF 1331 kb) [file 40560_2019_363_MOESM10_ESM.tif]

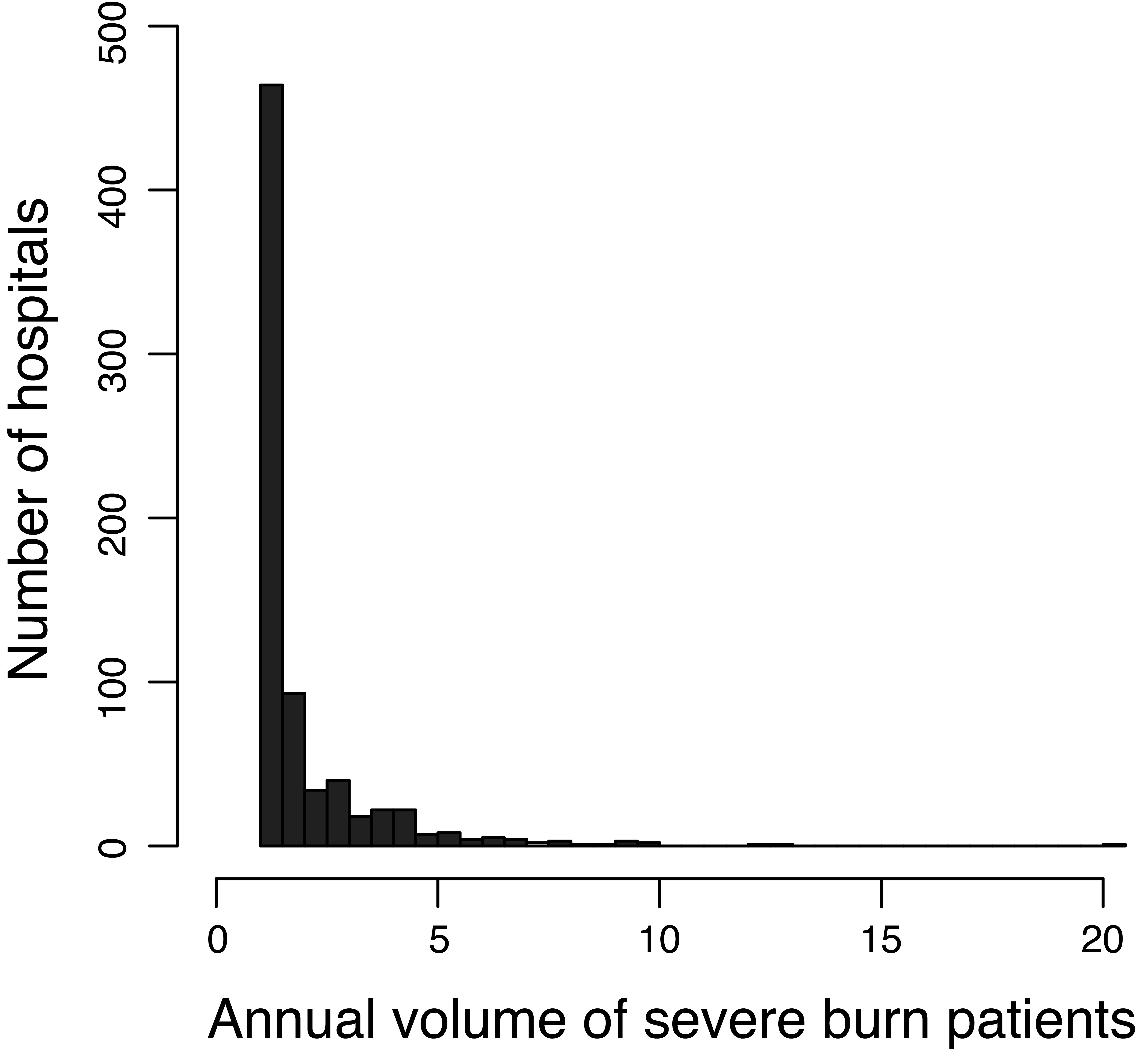

Supplement: Supplementary file 11 — Figure S6. Histogram of the hospital numbers and the volume of severe burn patients who survived for more than 2 days of admission. (TIF 1103 kb) [file 40560_2019_363_MOESM11_ESM.tif]

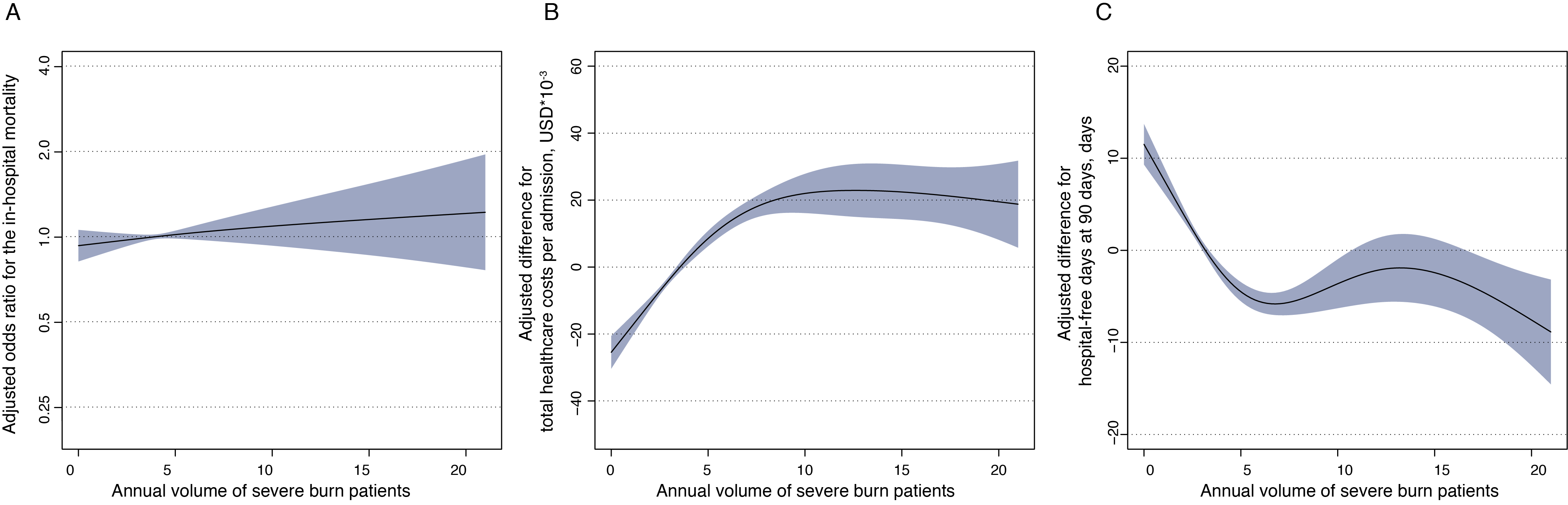

Supplement: Supplementary file 14 — Figure S7. Association between annual severe burn patient volume and the adjusted risk of (A) in-hospital survival, (B) total healthcare costs, and (C) hospital-free days at 90 days among patients who survived for more than 2 days of admission. The shaded region represents the standard errors for the point estimates. Patient severity was adjusted by the developed risk adjustment model as a fixed effect variable. The hospital unique identifier was also adjusted as a random effect variable. Abbreviation: USD, US dollars. (TIF 1282 kb) [file 40560_2019_363_MOESM14_ESM.tif]

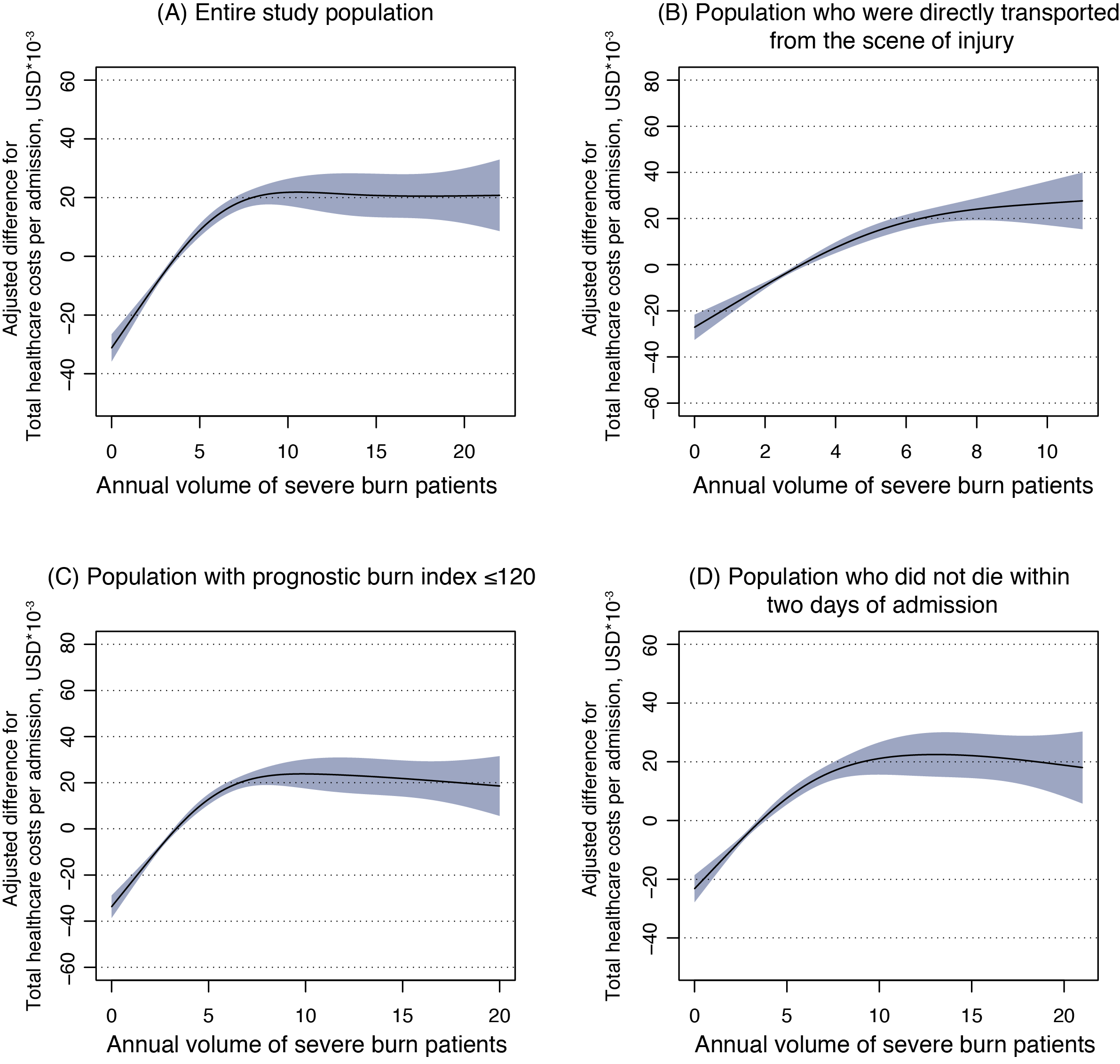

Supplement: Supplementary file 15 — Figure S8. Association between the annual severe burn patient volume and the adjusted difference of total healthcare costs with additional adjustment for the length of hospital stay in the (A) entire study population, (B) population who were directly transported from the scene of injury, (C) population with prognostic burn index ≤ 120, and (D) population excluding patients who died within 2 days of admission. The shaded region represents the standard errors for the point estimates. Patient severity was adjusted by the prognostic burn index in (A), (B), and (C) and by the developed risk adjustment model in (D). The hospital unique identifier was also adjusted as a random effect variable. Abbreviation: USD, US dollars. (TIF 1590 kb) [file 40560_2019_363_MOESM15_ESM.tif]
