# Supplementary material for: Volume-outcome relationship on survival and cost benefits in severe burn injury: a retrospective analysis of a Japanese nationwide administrative database
Source: J Intensive Care. 2019 Jan 30;7:7. doi: 10.1186/s40560-019-0363-7 (PMC6354429; doi:10.1186/s40560-019-0363-7)
Supplement: Supplementary file 4 — Table S2. Characteristics of the severe burn patients who were directly transported from the scene of injury (multiple imputed data). (DOCX 18 kb) [file 40560_2019_363_MOESM4_ESM.docx]

| **Supplementary Table 2. Patients’ characteristics in severe burn patients who were directly transported from the scene of injury (multiple imputed data)** | | | |
| --- | --- | --- | --- |
| Variables | | Annual severe burn patients ≤5 | Annual severe burn patients >5 |
| Number of hospitals, n | | 605 | 20 |
| Number of patients, n | | 3033 | 679 |
| Transferred from another hospital, n (%) | | 0 (0) | 0 (0) |
| Year of injury | |  |  |
|  | 2010–2012 | 1461 (48.2) | 320 (47.1) |
|  | 2013–2015 | 1572 (51.8) | 359 (52.9) |
| Age, years, median [IQR] | | 66 [43, 80] | 62 [41, 77] |
| Female sex, n (%) | | 1240 (40.9) | 252 (37.1) |
| Charlson comorbidity index, median (IQR) | | 0 [0, 0] | 0 [0, 0] |
| Levels of consciousness, alert, n (%) | | 1910 (63.0) | 390 (57.4) |
| Burn index, median (IQR) | | 15 [11, 27] | 20 [13.5, 40] |
| Prognostic burn index, median (IQR) | | 86.5 [64, 102] | 90 [66, 107] |
| Inhalation injury, n (%) | | 548 (18.1) | 164 (24.2) |
| Interventions performed within 2 days of admission | | | |
|  | Intensive care unit, n (%) | 1922 (63.3) | 580 (85.4) |
|  | Mechanical ventilation, n (%) | 1020 (33.6) | 345 (50.8) |
|  | Escharotomy, n (%) | 246 (8.1) | 115 (16.9) |
|  | Vasopressor, n (%) | 517 (17.0) | 140 (20.6) |
|  | Haptoglobin, n (%) | 299 (9.9) | 126 (18.6) |
|  | RBC transfusion, n (%) | 181 (6.0) | 41 (6.0) |
| Skin transplant during hospitalization, n (%) | | 1321 (43.6) | 359 (52.9) |
|  | Artificial graft use, n (%) | 294 (9.7) | 120 (17.7) |
|  | Cultured graft use, n (%) | 105 (3.5) | 65 (9.6) |
| Hospital characteristics | |  |  |
|  | A government-approved advanced hospital, n (%) | 717 (23.6) | 321 (47.3) |
|  | Number of ICU bed, median (IQR) | 3.8 [0, 6.5] | 4.9 [3.7, 11.3] |
| Abbreviation: IQR, interquartile range; ICU, intensive care unit | | | |
