# Supplementary material for: Volume-outcome relationship on survival and cost benefits in severe burn injury: a retrospective analysis of a Japanese nationwide administrative database
Source: J Intensive Care. 2019 Jan 30;7:7. doi: 10.1186/s40560-019-0363-7 (PMC6354429; doi:10.1186/s40560-019-0363-7)
Supplement: Supplementary file 8 — Table S4. Characteristics of the severe burn patients with prognostic burn index ≤ 120 (multiple imputed data). (DOCX 19 kb) [file 40560_2019_363_MOESM8_ESM.docx]

| **Supplementary Table 4. Patients’ characteristics in severe burn patients with prognostic burn index ≤120 (multiple imputed data)** | | | |
| --- | --- | --- | --- |
| Variables | | Annual severe burn patients ≤5 | Annual severe burn patients >5 |
| Number of hospitals, n | | 697 | 32 |
| Number of patients, n | | 3610 | 1156 |
| Transferred from another hospital, n (%) | | 1060 (29.4) | 396 (34.3) |
| Year of injury | |  |  |
|  | 2010–2012 | 1710 (47.4) | 516 (44.6) |
|  | 2013–2015 | 1900 (52.6) | 640 (55.4) |
| Age, years, median [IQR] | | 65.5 [41, 79] | 62.5 [39.75, 78] |
| Female sex, n (%) | | 1480 (41.0) | 463 (40.1) |
| Charlson comorbidity index, median (IQR) | | 0 [0, 1] | 0 [0, 0] |
| Levels of consciousness, alert, n (%) | | 2505 (69.4) | 696 (60.2) |
| Burn index, median (IQR) | | 15 [10.5, 20.5] | 17.5 [12, 28] |
| Prognostic burn index, median (IQR) | | 84 [61, 97] | 85 [62.5, 99.5] |
| Inhalation injury, n (%) | | 533 (14.8) | 238 (20.6) |
| Interventions performed within 2 days of admission | | | |
|  | Intensive care unit, n (%) | 1993 (55.2) | 965 (83.4) |
|  | Mechanical ventilation, n (%) | 907 (25.1) | 489 (42.3) |
|  | Escharotomy, n (%) | 187 (5.2) | 155 (13.4) |
|  | Vasopressor, n (%) | 479 (13.3) | 232 (20.0) |
|  | Haptoglobin, n (%) | 204 (5.7) | 160 (13.9) |
|  | RBC transfusion, n (%) | 179 (5.0) | 82 (7.1) |
| Skin transplant during hospitalization, n (%) | | 1664 (46.1) | 705 (61.0) |
|  | Artificial graft use, n (%) | 329 (9.1) | 214 (18.5) |
|  | Cultured graft use, n (%) | 114 (3.1) | 96 (8.3) |
| Hospital characteristics | |  |  |
|  | A government-approved advanced hospital, n (%) | 868 (24.0) | 514 (44.5) |
|  | Number of ICU bed, median (IQR) | 3.7 [0, 6.5] | 4.9 [3.7, 9.6] |
|  | Proportion of transferred patients of a treating hospital, median (IQR) | 25.0 [11.1, 42.3] | 30.2 [17.9, 47.2] |
| Abbreviation: IQR, interquartile range; ICU, intensive care unit | | | |
