# Supplementary material for: Volume-outcome relationship on survival and cost benefits in severe burn injury: a retrospective analysis of a Japanese nationwide administrative database
Source: J Intensive Care. 2019 Jan 30;7:7. doi: 10.1186/s40560-019-0363-7 (PMC6354429; doi:10.1186/s40560-019-0363-7)
Supplement: Supplementary file 9 — Table S5. Characteristics of the severe burn patients who survived for patients with prognostic burn index ≤ 120 (naïve data). (DOCX 19 kb) [file 40560_2019_363_MOESM9_ESM.docx]

| **Supplementary Table 5. Patients’ characteristics in severe burn patients with prognostic burn index ≤120 (naïve data)** | | | | | |
| --- | --- | --- | --- | --- | --- |
| Variables | | Annual severe burn patients ≤5 | | Annual severe burn patients >5 | |
|  |  | Registered | Missing, n (%) | Registered | Missing, n (%) |
| Number of hospitals, n | | 697 | 0 (0) | 32 | 0 (0) |
| Number of patients, n | | 3610 | 0 (0) | 1156 | 0 (0) |
| Transferred from another hospital, n (%) | | 1060 (29.4) | 0 (0) | 396 (34.3) | 0 (0) |
| Year of injury | |  | 0 (0) |  | 0 (0) |
|  | 2010–2012 | 1710 (47.4) | - | 516 (44.6) | - |
|  | 2013–2015 | 1900 (52.6) | - | 640 (55.4) | - |
| Age, years, median [IQR] | | 65.5 [41, 79] | 0 (0) | 62.5 [39.75, 78] | 0 (0) |
| Female sex, n (%) | | 1480 (41.0) | 0 (0) | 463 (40.1) | 0 (0) |
| Charlson comorbidity index, median (IQR) | | 0 [0, 1] | 0 (0) | 0 [0, 0] | 0 (0) |
| Levels of consciousness, alert, n (%) | | 2505 (69.4) | 0 (0) | 696 (60.2) | 0 (0) |
| Burn index, median (IQR) | | 15 [10.5, 20.5] | 0 (0) | 17.5 [12, 28] | 0 (0) |
| Prognostic burn index, median (IQR) | | 84 [61, 97] | 0 (0) | 85 [62.5, 99.5] | 0 (0) |
| Inhalation injury, n (%) | | 533 (14.8) | 0 (0) | 238 (20.6) | 0 (0) |
| Interventions performed within 2 days of admission | | | | | |
|  | Intensive care unit, n (%) | 1954 (54.1) | 248 (6.9) | 956 (82.7) | 37 (3.2) |
|  | Mechanical ventilation, n (%) | 902 (25.0) | 248 (6.9) | 487 (42.1) | 37 (3.2) |
|  | Escharotomy, n (%) | 183 (5.1) | 248 (6.9) | 154 (13.3) | 37 (3.2) |
|  | Vasopressor, n (%) | 474 (13.2) | 248 (6.9) | 231 (20.0) | 37 (3.2) |
|  | Haptoglobin, n (%) | 203 (5.6) | 248 (6.9) | 160 (13.8) | 37 (3.2) |
|  | RBC transfusion, n (%) | 176 (4.9) | 248 (6.9) | 82 (7.1) | 37 (3.2) |
| Skin transplant during hospitalization, n (%) | | 1624 (45.0) | 248 (6.9) | 700 (60.6) | 37 (3.2) |
|  | Artificial graft use, n (%) | 326 (9.0) | 248 (6.9) | 214 (18.5) | 37 (3.2) |
|  | Cultured graft use, n (%) | 113 (3.1) | 248 (6.9) | 96 (8.3) | 37 (3.2) |
| Hospital characteristics | | | | | |
|  | A government-approved advanced hospital, n (%) | 868 (24.0) | 0 (0) | 514 (44.5) | 0 (0) |
|  | Number of ICU bed, median (IQR) | 3.7 [0, 6.5] | 0 (0) | 4.9 [3.7, 9.6] | 0 (0) |
|  | Proportion of transferred patients of a treating hospital, median (IQR) | 25.0 [11.1, 42.3] | 0 (0) | 30.2 [17.9, 47.2] | 0 (0) |
| Abbreviation: IQR, interquartile range; ICU, intensive care unit | | | | | |
