# Supplementary material for: Volume-outcome relationship on survival and cost benefits in severe burn injury: a retrospective analysis of a Japanese nationwide administrative database
Source: J Intensive Care. 2019 Jan 30;7:7. doi: 10.1186/s40560-019-0363-7 (PMC6354429; doi:10.1186/s40560-019-0363-7)
Supplement: Supplementary file 12 — Table S6. Characteristics of the severe burn patients who survived for more than 2 days of admission (multiple imputed data) (DOCX 19 kb) [file 40560_2019_363_MOESM12_ESM.docx]

| **Supplementary Table 6. Patients’ characteristics in severe burn patients who did not die within two days of admission (multiple imputed data)** | | | |
| --- | --- | --- | --- |
| Variables | | Annual severe burn patients ≤5 | Annual severe burn patients >5 |
| Number of hospitals, n | | 700 | 36 |
| Number of patients, n | | 3587 | 1332 |
| Transferred from another hospital, n (%) | | 1034 (28.8) | 459 (34.5) |
| Year of injury | |  |  |
|  | 2010–2012 | 1694 (47.2) | 614 (46.1) |
|  | 2013–2015 | 1893 (52.8) | 718 (53.9) |
| Age, years, median [IQR] | | 66 [42, 80] | 64 [42, 78] |
| Female sex, n (%) | | 1483 (41.3) | 527 (39.6) |
| Charlson comorbidity index, median (IQR) | | 0 [0, 1] | 0 [0, 0] |
| Levels of consciousness, alert, n (%) | | 2494 (69.5) | 809 (60.7) |
| Burn index, median (IQR) | | 15 [10.5, 22.5] | 18 [12.5, 31] |
| Prognostic burn index, median (IQR) | | 85 [62.5, 98] | 87 [65, 102] |
| Inhalation injury, n (%) | | 529 (14.7) | 275 (20.6) |
| Interventions performed within 2 days of admission | | | |
|  | Intensive care unit, n (%) | 1972 (55.0) | 1112 (83.5) |
|  | Mechanical ventilation, n (%) | 915 (25.5) | 580 (43.5) |
|  | Escharotomy, n (%) | 218 (6.1) | 199 (14.9) |
|  | Vasopressor, n (%) | 482 (13.4) | 276 (20.7) |
|  | Haptoglobin, n (%) | 242 (6.7) | 204 (15.3) |
|  | RBC transfusion, n (%) | 190 (5.3) | 102 (7.7) |
| Skin transplant during hospitalization, n (%) | | 1669 (46.5) | 822 (61.7) |
|  | Artificial graft use, n (%) | 351 (9.8) | 254 (19.1) |
|  | Cultured graft use, n (%) | 123 (3.4) | 121 (9.1) |
| Hospital characteristics | |  |  |
|  | A government-approved advanced hospital, n (%) | 845 (23.6) | 596 (44.7) |
|  | Number of ICU bed, median (IQR) | 3.6 [0, 6.4] | 4.9 [3.7, 9.5] |
|  | Proportion of transferred patients of a treating hospital, median (IQR) | 25.0 (11.1, 41.2) | 31.5 (18.4, 50.0) |
| Abbreviation: IQR, interquartile range; ICU, intensive care unit | | | |
