# Supplementary material for: Volume-outcome relationship on survival and cost benefits in severe burn injury: a retrospective analysis of a Japanese nationwide administrative database
Source: J Intensive Care. 2019 Jan 30;7:7. doi: 10.1186/s40560-019-0363-7 (PMC6354429; doi:10.1186/s40560-019-0363-7)
Supplement: Supplementary file 13 — Table S7. Characteristics of the severe burn patients who survived for patients who survived for more than 2 days of admission (naïve data). (DOCX 19 kb) [file 40560_2019_363_MOESM13_ESM.docx]

| **Supplementary Table 7. Patients’ characteristics in s severe burn patients who did not die within two days of admission (naïve data)** | | | | | |
| --- | --- | --- | --- | --- | --- |
| Variables | | Annual severe burn patients ≤5 | | Annual severe burn patients >5 | |
|  |  | Registered | Missing, n (%) | Registered | Missing, n (%) |
| Number of hospitals, n | | 700 | 0 (0) | 36 | 0 (0) |
| Number of patients, n | | 3587 | 0 (0) | 1332 | 0 (0) |
| Transferred from another hospital, n (%) | | 1034 (28.8) | 0 (0) | 459 (34.5) | 0 (0) |
| Year of injury | |  | 0 (0) |  | 0 (0) |
|  | 2010–2012 | 1694 (47.2) | - | 614 (46.1) | - |
|  | 2013–2015 | 1893 (52.8) | - | 718 (53.9) | - |
| Age, years, median [IQR] | | 66 [42, 80] | 0 (0) | 64 [42, 78] | 0 (0) |
| Female sex, n (%) | | 1483 (41.3) | 0 (0) | 527 (39.6) | 0 (0) |
| Charlson comorbidity index, median (IQR) | | 0 [0, 1] | 0 (0) | 0 [0, 0] | 0 (0) |
| Levels of consciousness, alert, n (%) | | 2494 (69.5) | 0 (0) | 809 (60.7) | 0 (0) |
| Burn index, median (IQR) | | 15 [10.5, 22.5] | 0 (0) | 18 [12.5, 31] | 0 (0) |
| Prognostic burn index, median (IQR) | | 85 [62.5, 98] | 0 (0) | 87 [65, 102] | 0 (0) |
| Inhalation injury, n (%) | | 529 (14.7) | 0 (0) | 275 (20.6) | 0 (0) |
| Interventions performed within 2 days of admission | | | | | |
|  | Intensive care unit, n (%) | 1935 (53.9) | 245 (6.8) | 1099 (82.5) | 47 (3.5) |
|  | Mechanical ventilation, n (%) | 910 (25.4) | 245 (6.8) | 577 (43.3) | 47 (3.5) |
|  | Escharotomy, n (%) | 214 (6.0) | 245 (6.8) | 198 (14.9) | 47 (3.5) |
|  | Vasopressor, n (%) | 479 (13.4) | 245 (6.8) | 275 (20.6) | 47 (3.5) |
|  | Haptoglobin, n (%) | 241 (6.7) | 245 (6.8) | 203 (15.2) | 47 (3.5) |
|  | RBC transfusion, n (%) | 187 (5.2) | 245 (6.8) | 101 (7.6) | 47 (3.5) |
| Skin transplant during hospitalization, n (%) | | 1628 (45.4) | 245 (6.8) | 814 (61.1) | 47 (3.5) |
|  | Artificial graft use, n (%) | 348 (9.7) | 245 (6.8) | 253 (19.0) | 47 (3.5) |
|  | Cultured graft use, n (%) | 122 (3.4) | 245 (6.8) | 121 (9.1) | 47 (3.5) |
| Hospital characteristics | | | | | |
|  | A government-approved advanced hospital, n (%) | 845 (23.6) | 0 (0) | 596 (44.7) | 0 (0) |
|  | Number of ICU bed, median (IQR) | 3.6 [0, 6.4] | 0 (0) | 4.9 [3.7, 9.5] | 0 (0) |
|  | Proportion of transferred patients of a treating hospital, median (IQR) | 25.0 (11.1, 41.2) | 0 (0) | 31.5 (18.4, 50.0) | 0 (0) |
| Abbreviation: IQR, interquartile range; ICU, intensive care unit | | | | | |
