# Supplementary material for: Volume-outcome relationship on survival and cost benefits in severe burn injury: a retrospective analysis of a Japanese nationwide administrative database
Source: J Intensive Care. 2019 Jan 30;7:7. doi: 10.1186/s40560-019-0363-7 (PMC6354429; doi:10.1186/s40560-019-0363-7)
Supplement: Supplementary file 16 — Table S8. Results of the generalized linear mixed-effect model analysis for total healthcare costs with additional adjustment for the length of hospital stay. (DOCX 15 kb) [file 40560_2019_363_MOESM16_ESM.docx]

| **Supplementary Table 8. Results of the generalized linear mixed-effect model analysis for total healthcare costs with additional adjustment for the length of hospital stay** | | | |
| --- | --- | --- | --- |
| **Total healthcare costs per admission, USD** | | **Adjusted difference**  **(95% CI)** | ***p*-value** |
| **Models in which patient volume was included as a continuous variable** | | | |
|  | Entire study population | 4643 (4224 – 5061) | < 0.001 |
|  | Population who were directly transported from the scene of injury | 6587 (5936 – 7238) | < 0.001 |
|  | Population with prognostic burn index ≤120 | 5696 (5202 – 6190) | < 0.001 |
|  | Population excluding patients who died within 2 days of admission | 6149 (5377 – 6921) | < 0.001 |
| **Models in which patient volume was included as a categorical variable** | | | |
|  | Entire study population | 28,451 (25,294 – 31,607) | < 0.001 |
|  | Population who were directly transported from the scene of injury | 24,240 (19,967 – 28,513) | < 0.001 |
|  | Population with prognostic burn index ≤120 | 28,723 (25,026 – 32,420)) | < 0.001 |
|  | Population excluding patients who died within 2 days of admission | 21,660 (16,030 – 27,290) | < 0.001 |
| Patient severity was adjusted by the prognostic burn index in the entire study population and the population who were directly transported from the scene of injury, and by the developed risk adjustment model in the population excluding patients who died within 2 days of admission. The hospital unique identifier was also adjusted as a random effect variable. Abbreviations: USD, US dollars; CI, confidence interval. | | | |
